# A plasma proteomics-based candidate biomarker panel predictive of amyotrophic lateral sclerosis

**DOI:** 10.1038/s41591-025-03890-6

**Published:** 2025-08-19

**Authors:** Ruth Chia, Ruin Moaddel, Justin Y. Kwan, Memoona Rasheed, Paola Ruffo, Natalie Landeck, Paolo Reho, Rosario Vasta, Andrea Calvo, Cristina Moglia, Antonio Canosa, Umberto Manera, Allison Snyder, Sara Saez-Atienzar, Maurizio Grassano, Maura Brunetti, Federico Casale, Anindita Ray, Kumar Arvind, Betul Comertpay, Min Zhu, J. Raphael Gibbs, The American Genome Center, Camille Alba, Ted M. Dawson, Liana S. Rosenthal, Anna J. Hall, Alexander Y. Pantelyat, Derek P. Narendra, Debra J. Ehrlich, Keenan A. Walker, Peter Kosa, Bibiana Bielekova, Josephine M. Egan, Julián Candia, Toshiko Tanaka, Luigi Ferrucci, Clifton L. Dalgard, Sonja W. Scholz, Adriano Chiò, Bryan J. Traynor

**Affiliations:** 1 Neuromuscular Diseases Research Section, National Institute on Aging, National Institutes of Health, Bethesda, MD USA; 2 Laboratory of Clinical Investigation, National Institute on Aging, National Institutes of Health, Baltimore, MD USA; 3 Neurodegenerative Disorders Clinic, National Institute of Neurological Disorders and Stroke, National Institutes of Health, Bethesda, MD USA; 4 Neurodegenerative Diseases Research Section, National Institute of Neurological Disorders and Stroke, National Institutes of Health, Bethesda, MD USA; 5 Department of Environmental Health Sciences, Mailman School of Public Health, Columbia University, New York, NY USA; 6 ‘Rita Levi Montalcini’ Department of Neuroscience, Amyotrophic Lateral Sclerosis Center, University of Turin, Turin, Italy; 7 Azienda Ospedaliero Universitaria Città della Salute e della Scienza, Turin, Italy; 8 Institute of Cognitive Sciences and Technologies, C.N.R, Rome, Italy; 9 Department of Neurology, The Ohio State University, Columbus, OH USA; 10 Computational Biology Group, Laboratory of Neurogenetics, National Institute on Aging, National Institutes of Health, Bethesda, MD USA; 11 Department of Anatomy, Physiology and Genetics, Uniformed Services University of the Health Sciences, Bethesda, MD USA; 12 The American Genome Center, Uniformed Services University of the Health Sciences, Bethesda, MD USA; 13 Henry M. Jackson Foundation for the Advancement of Military Medicine, Bethesda, MD USA; 14 Neuroregeneration and Stem Cell Programs, Institute for Cell Engineering, Johns Hopkins University School of Medicine, Baltimore, MD USA; 15 Department of Neurology, Johns Hopkins University School of Medicine, Baltimore, MD USA; 16 Inherited Movement Disorders Unit, Neurogenetics Branch, National Institute of Neurological Disorders and Stroke, National Institutes of Health, Bethesda, MD USA; 17 Parkinson’s Disease Clinic, National Institute of Neurological Disorders and Stroke, National Institutes of Health, Bethesda, MD USA; 18 Laboratory of Behavioral Neuroscience, National Institute on Aging, National Institutes of Health, Baltimore, MD USA; 19 Laboratory of Clinical Immunology and Microbiology, Neuroimmunological Diseases Section, National Institute of Allergy and Infectious Diseases, National Institutes of Health, Bethesda, MD USA; 20 Longitudinal Studies Section, National Institute on Aging, National Institutes of Health, Baltimore, MD USA; 21 National Institute of Neurological Disorders and Stroke, National Institutes of Health, Bethesda, MD USA; 22 RNA Therapeutics Laboratory, National Center for Advancing Translational Sciences, National Institutes of Health, Rockville, MD USA

**Keywords:** Diagnostic markers, Predictive markers

## Abstract

Identifying a reliable biomarker for amyotrophic lateral sclerosis (ALS) is crucial for clinical practice. Here, in this cross-sectional study, we used the Olink Explore 3072 platform to investigate plasma proteomics as a biomarker tool for this neurodegenerative condition. Thirty-three proteins were differentially abundant in the plasma of patients with ALS (*n* = 183) versus controls (*n* = 309). We replicated our findings in an independent cohort (*n* = 48 patients with ALS and *n* = 75 controls). We then applied machine learning to create a model that diagnosed ALS with high accuracy (area under the curve, 98.3%). By analyzing plasma samples from individuals before ALS symptoms emerged, we estimated the age of clinical onset and showed that the disease process—impacting skeletal muscle, nerves and energy metabolism—occurs years before symptoms appear. Our research suggests that plasma proteins can be a biomarker for this fatal disease and offers molecular insights into its prodromal phase.

## Abstract (web summary)

Plasma proteomic analysis of presymptomatic individuals with amyotrophic lateral sclerosis identifies novel disease-associated proteins and enables the development of an accurate diagnostic biomarker panel.

## Main

Amyotrophic lateral sclerosis (ALS) is a progressive neurological disorder characterized by the degeneration of motor neurons. This degeneration leads to muscle weakness, atrophy and, ultimately, respiratory failure, typically resulting in death within 2–4 years after symptoms begin^1^. By 2040, the global population of individuals living with ALS worldwide is projected to approach 400,000 (ref. ^2^). The substantial number of cases and the high cost associated with their care pose a substantial burden on communities and healthcare systems^2^. In the United States, the only approved treatments for sporadic ALS are riluzole and edaravone. There is an urgent need for better medications; however, the necessity to recruit large numbers of patients with ALS into clinical trials to assess drug effectiveness hinders therapeutic development^3^.

Currently, diagnosing ALS relies on clinical symptoms and neurological evaluations, much like the methods used 155 years ago when Jean-Martin Charcot first identified the disease^4^. Although neurophysiological studies and genetic tests are supportive tools for clinical decision-making, no definitive diagnostic test exists^5^. Patients frequently experience delays of 6–18 months until their symptoms become severe enough to establish a diagnosis^6^. Even when there is clear evidence of motor neuron dysfunction, differentiating ALS from other neurological conditions can be difficult because the disease presents with heterogeneous clinical manifestations^7^. This uncertainty can be unsettling for patients and their families. Moreover, it causes delays in entering clinical trials and limits the number of eligible participants due to enrollment restrictions for patients with more severe disability^8^. In the future, when effective treatments become available for ALS, diagnostic delays may impede treatment initiation, harming patient outcomes.

We addressed this critical knowledge gap by generating and analyzing data for 3,072 plasma proteins within a large ALS case–control cohort. These well-characterized patients had genetic data readily available, allowing us to incorporate these factors into our search for differentially abundant proteins and to use a multiomic approach to determine if the observed plasma protein changes occurred because of the disease process or were primary drivers in the pathogenesis of ALS. Using machine learning, we identified a distinct molecular signature in plasma that effectively differentiates ALS cases from healthy individuals and other neurological disorders. Additionally, we explored whether our model could predict when symptoms would start in asymptomatic individuals who later developed ALS. Notably, we established a public resource for the scientific community to encourage and advance biomarker research in this fatal neurodegenerative disease.

## Results

After quality control, we obtained data for 2,886 proteins from 231 individuals diagnosed with ALS and 384 control individuals (*n* = 214 healthy individuals and *n* = 170 with other neurological conditions). Patients with other neurological disorders were included as control data, as our central hypothesis was to use proteomics to distinguish ALS cases both from healthy individuals and from other relevant conditions. Participants were recruited from an Italian population-based ALS registry and a US natural history study, and they were diagnosed with ALS based on established consensus criteria^9,10^. Extended Data Table 1 lists the demographics and clinical features of the cohorts used in this study.

**Fig. 1:**
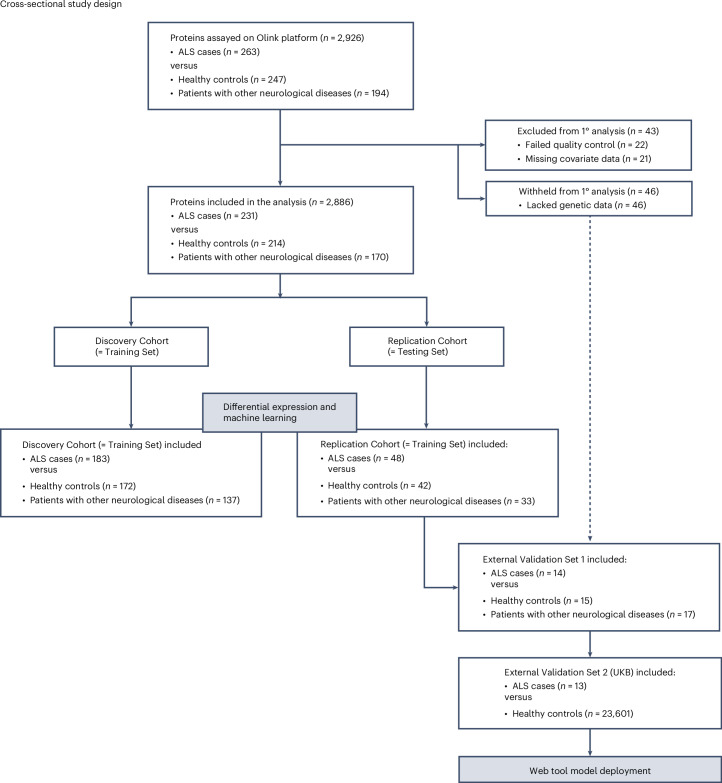
Study workflow. We conducted a cross-sectional study to identify ALS biomarkers via proteomic analysis of plasma samples. We performed differential abundance analysis on proteomic data generated from plasma samples of patients with ALS, healthy controls and patients with other neurological diseases in both the Discovery Cohort and the Replication Cohort. The other neurological diseases included corticobasal syndrome (*n* = 8 patients), Lewy body dementia (*n* = 8 patients), multiple system atrophy (*n* = 5 patients), Parkinson’s disease (*n* = 153 patients), progressive supranuclear palsy (*n* = 19 patients) and dementia, not otherwise specified (*n* = 1 patient). After this, we applied supervised machine learning using plasma protein levels and clinical parameters to identify a molecular signature of ALS. The same samples from the Discovery Cohort and the Replication Cohort sequentially formed the Training Set and the Testing Set in the machine learning process. The 46 samples withheld from the initial analyses due to the absence of genetic data were labeled as External Validation Set 1. For External Validation Set 2, we obtained proteomic data from the UK Biobank^18^. Additionally, a web tool was created for clinical researchers to analyze their own data. ‘Neurological’ pertains to neurological conditions other than ALS. 1°, first degree; UKB, UK Biobank.

The proteomic data, measured using the Olink Explore 3072 assay in plasma samples, exhibited median intra-assay and inter-assay coefficients of variation of 9.9% and 22.3%, respectively, confirming the platform’s reliability^11^. Figure 1 gives an overview of the study and analysis. We randomly designated 80% of the samples as the Discovery Cohort (*n* = 183 ALS cases versus *n* = 172 healthy controls plus *n* = 137 other neurological diseases). The remaining samples constituted the Replication Cohort (*n* = 48 ALS cases versus *n* = 42 healthy controls plus *n* = 33 other neurological diseases).

### Proteome-wide association analysis reveals plasma proteins linked to ALS

We conducted proteome-wide association testing of individual plasma protein levels using generalized linear regression. Our analyses were adjusted for the following confounding factors: age at sample collection, self-reported sex and the collection tube type (that is, EDTA or heparin). Furthermore, we incorporated the first two uniform manifold approximation and projection (UMAP) components derived from genetic data of the study participants to manage population stratification.

**Fig. 2:**
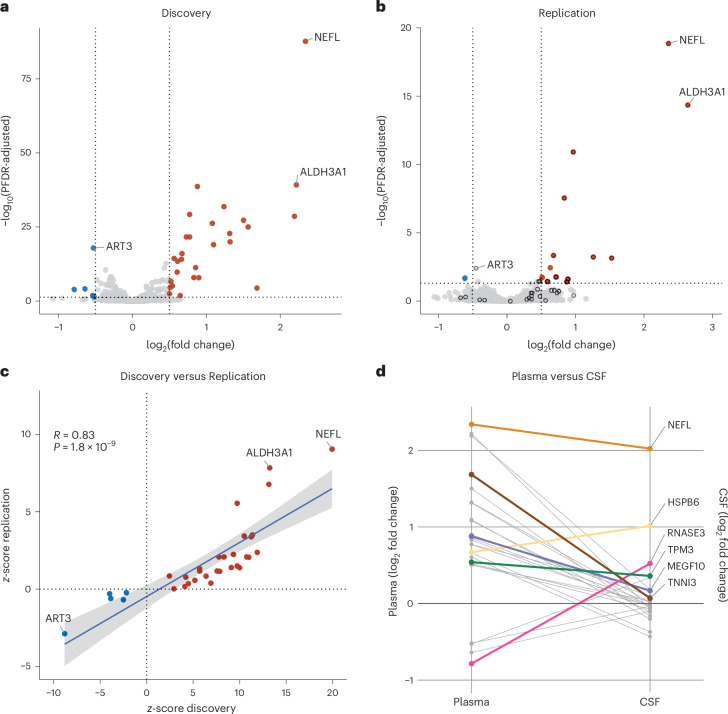
Differential abundance of plasma proteins in patients diagnosed with ALS compared with control individuals. **a**, Volcano plot showing the differential abundance of proteins in the Discovery Cohort (*n* = 183 ALS cases versus *n* = 172 healthy controls plus *n* = 137 other neurological diseases). The dotted vertical lines delineate a ±1.4-fold change threshold, and the dotted horizontal lines represent the 0.05 *P*-value threshold. Blue and red dots denote statistically significant downregulated or upregulated proteins determined by generalized linear regression (adjusted to 5% FDR for multiple comparisons). **b**, Volcano plot showing the differential abundance of proteins in the Replication Cohort (*n* = 48 ALS cases versus *n* = 42 healthy controls and *n* = 33 other neurological diseases). Black circles highlight the proteins that were significant in the Discovery Cohort. **c**, Scatter plot comparing the discovery and replication *z*-scores of the 33 proteins significantly associated with ALS in the Discovery Cohort. The error band is shown as a gray band representing the 95% confidence interval for the mean prediction (blue linear regression line) at each *x* value. Pearson correlation coefficients (R) were calculated to assess the linear association between variables. *P* values were computed using a two-sided test of the null hypothesis that the correlation coefficient equals 0. **d**, A comparison of the proteins significantly associated with ALS in plasma and their abundances in CSF. The CSF protein levels were derived from SomaScan data generated for *n* = 14 ALS cases and *n* = 89 healthy controls. The 6 proteins significantly correlated with ALS in the CSF are highlighted in color, whereas the other 21 appear in gray. The additional six proteins were not tested by the SomaScan platform. The *y* axis indicates a log_2_(fold change) relative to the control cohort.

In total, 33 plasma proteins exceeded the significance threshold in the Discovery Cohort (adjusted false discovery rate (FDR) *P* < 0.05; Table 1 and Fig. 2a). The protein with the highest differential protein abundance was neurofilament light chain (NEFL, log_2_ fold change = 2.34, adjusted FDR *P* = 2.22 × 10^−88^), which is known to be elevated in ALS and various neurological diseases^12^. Notably, LIF encodes leukemia inhibitory factor, a neurotrophic cytokine that may promote motor neuron survival in vivo and in vitro^13^. The remaining 31 proteins have not been definitively linked to ALS previously.

**Table 1:** Proteins associated with ALS based on differential abundance analysis

Protein	Olink Explore 3072 (plasma)				SomaScan 7k (CSF)		
	Discovery Cohort		Replication Cohort		Cohort		
	log_2_ fold change	FDR-adjusted *P* value	log_2_ fold change	FDR-adjusted *P* value	Estimate	s.e.	*P* value
NEFL	2.34	2.22 × 10^−88^	2.36	**1.39 × 10**^**−19**^	2.02	0.11	**8.71 × 10**^**−35**^
ALDH3A1	2.22	6.27 × 10^−40^	2.64	**4.47 × 10**^**−15**^	−0.04	0.02	0.057
MEGF10	0.88	2.14 × 10^−39^	0.97	**1.22 × 10**^**−11**^	0.12	0.02	**5.34 × 10**^**−10**^
CORO6	1.24	1.39 × 10^−32^	0.72	**0.0174**	NA	NA	NA
HS6ST2	0.77	5.81 × 10^−30^	0.68	**4.61 × 10**^**−4**^	−0.02	0.02	0.44
CSRP3	2.19	2.56 × 10^−29^	1.53	**7.18 × 10**^**−4**^	0.11	0.10	0.25
MYBPC1	1.50	5.31 × 10^−28^	0.87	**0.0392**	−0.09	0.09	0.30
CA3	1.08	5.91 × 10^−27^	0.59	**0.0374**	0.36	0.32	0.27
MYLPF	1.56	1.01 × 10^−25^	1.26	**6.01 × 10**^**−4**^	NA	NA	NA
MYOM3	1.32	1.58 × 10^−23^	0.69	0.166	−0.03	0.02	0.10
RBFOX3	0.73	2.26 × 10^−22^	0.45	0.135	NA	NA	NA
EDA2R	0.77	2.34 × 10^−22^	0.84	**2.86 × 10**^**−8**^	0.17	0.07	0.02
MYBPC2	1.32	1.03 × 10^−20^	0.89	**0.0250**	0.01	0.01	0.48
ACTN2	1.10	9.45 × 10^−20^	0.65	0.163	−0.03	0.30	0.91
ART3	−0.53	1.22 × 10^−18^	−0.45	**3.98 × 10**^**−3**^	0.25	0.12	0.03
HSPB6	0.67	9.47 × 10^−17^	0.48	**0.0374**	0.85	0.11	**1.87 × 10**^**−12**^
DTNB	0.56	4.17 × 10^−15^	0.35	0.249	NA	NA	NA
MB	0.66	8.15 × 10^−15^	0.46	**0.0374**	−0.19	0.22	0.40
NEB	0.61	3.75 × 10^−14^	0.37	0.241	NA	NA	NA
MYL3	0.85	5.07 × 10^−12^	0.36	0.698	0.04	0.02	0.13
KLHL41	0.61	1.71 × 10^−10^	0.36	0.402	−0.29	0.14	0.03
DUSP29	0.83	1.20 × 10^−8^	0.73	0.241	−0.03	0.03	0.33
MYOM2	0.90	1.29 × 10^−8^	0.76	0.185	0.11	0.04	0.01
TTN	0.52	2.23 × 10^−7^	0.32	0.570	−0.02	0.02	0.36
TPM3	0.54	7.56 × 10^−6^	0.29	0.724	0.27	0.03	**8.39 × 10**^**−16**^
KLK4	0.51	2.59 × 10^−5^	0.48	0.437	−0.06	0.02	0.02
TNNI3	1.68	4.06 × 10^−5^	0.56	0.86	0.04	0.01	0.01
LEP	−0.64	6.89 × 10^−5^	−0.39	0.767	−0.15	0.10	0.15
RNASE3	−0.79	1.15 × 10^−4^	−0.67	0.543	0.40	0.06	**2.88 × 10**^**−9**^
LIF	0.50	3.31 × 10^−3^	0.05	0.986	0.02	0.02	0.42
SSC4D	−0.54	0.0125	−0.60	0.491	NA	NA	NA
FGF21	0.65	0.0143	0.97	0.395	NA	NA	NA
GZMH	−0.52	0.0298	−0.32	0.818	−0.03	0.01	0.01

The Discovery Cohort consisted of 183 ALS cases and 309 control individuals, and the Replication Cohort comprised 48 ALS cases and 75 control individuals. Plasma protein abundances in these samples were assayed using the Olink Explore 3072 platform. The abundances of proteins (*n* = 26) in the CSF from a separate cohort (*n* = 14 ALS cases and *n* = 89 healthy individuals) were assessed using the SomaScan 7K platform. Proteins that replicated (FDR-adjusted *P* < 0.05 for plasma proteins and *P* < 0.002 (0.05/26) for CSF proteins) are in bold. NA, not available; s.e., standard error of the estimate derived from generalized linear model.

**Extended Data Fig. 1:**
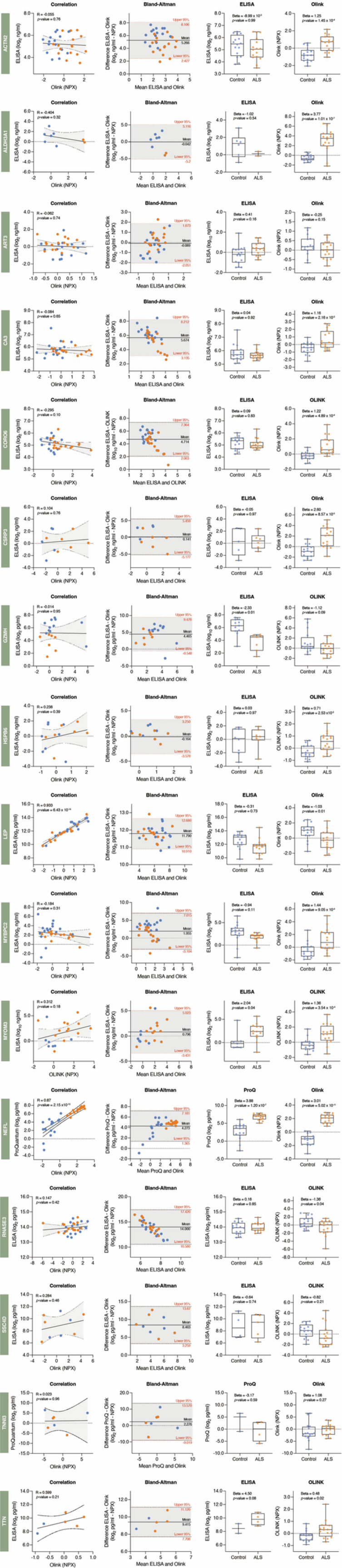
Comparison of proteins quantified using Olink and ELISA assays. ELISA assays were performed on plasma samples from the Discovery and Replication datasets (n = 16 ALS cases and n = 16 healthy controls). ELISA kits were available for 30 of the 33 differentially abundant proteins. We present exploratory data for 16 proteins, while the other ELISA assays are excluded due to either lack of protein detection (n = 13) or assay failure (n = 1). Each row displays data for an individual protein. The agreement between protein measurements from the ELISA or ProQuantum^TM^ (abbreviated as ProQ) and Olink assays was evaluated using three methods: correlation (first panel), Bland-Altman analysis (second panel), and differential analysis comparing ALS (orange dots) with healthy controls (blue dots) through either ELISA or ProQuantum^TM^ (third panel) or Olink (fourth panel) measurements. Pearson correlation coefficients (R) were calculated to assess the linear association between variables. P-values were computed using a two-sided test of the null hypothesis that the correlation coefficient equals zero. The grey area indicates the 95% confidence interval for the mean prediction (black linear regression line) at each x-value. In the Bland-Altman (B-A) plots, the mean bias difference between the two assays is depicted as a solid horizontal black line in the center of the plot. The shaded grey area reflects the 95% limits of agreement for the paired measurements from each sample, with upper and lower limit values shown in red font. The box-and-whisker plots in the differential analysis panels present the range (whiskers) alongside the first, median, and third quartiles of the measurements within the box. The effect size of the regression, or beta, along with the corresponding p-values, is included in each panel.

**Extended Data Fig. 2:**
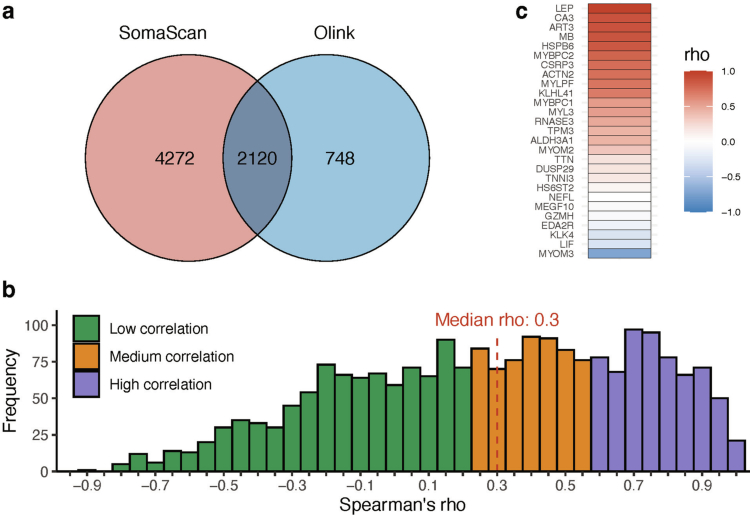
Comparison between SomaScan 7k and Olink 3072 Explore in BLSA samples. Plasma profiling was performed on a random subset of the Baltimore Longitudinal Study of Aging (BLSA) samples (*n* = 9) using the SomaScan 7 K and Olink 3072 Explore platforms. **a**, The Venn diagram shows the overlap of the UniProt targets assayed on the two platforms. Of the 2,886 proteins assayed on the Olink platform, there were 2,868 unique UniProt IDs. Of these, 2,120 proteins overlapped with the SomaScan 7 K platform. **b**, Spearman correlations between the overlapping protein measurements on the Olink and SomaScan platforms. K-means clustering, defined by Katz and colleagues, divided the distribution into three clusters. **c**, The heatmap illustrates Spearman correlations among overlapping protein measurements from the Olink and SomaScan platforms, specifically for 27 differentially expressed proteins in ALS cases. Data for the six other differentially expressed proteins (CORO6, DTNB, FGF21, NEB, RBFOX3, and SSC4D) were unavailable.

We verified the differential abundance of the proteins (*n* = 14 of 33) identified in the Discovery Cohort within the independent Replication Cohort, which similarly included ALS cases, healthy controls and individuals with other neurological diseases (Table 1 and Fig. 2b). The other proteins (*n* = 9) also showed the same directional effect observed in the Discovery Cohort (Table 1), leading to a high concordance of 0.83 between the discovery and replication analyses for the 33 proteins (Fig. 2c; *R* = 0.83, *P* = 1.80 × 10^−9^). Additional validation was performed using sensitivity analyses (Extended Data Table 2), comparisons with quantitative ELISAs (Extended Data Fig. 1) and analyses against SomaScan 7K proteomic data (Extended Data Fig. 2).

### Comparative analysis of plasma biomarkers in patients with *C9orf72* ALS

**Extended Data Fig. 3:**
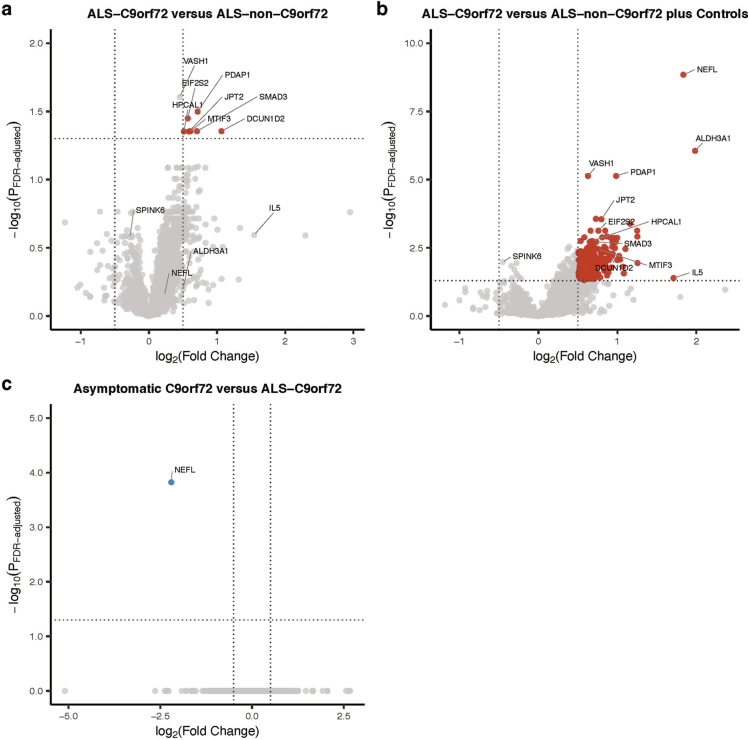
Differential abundance of plasma proteins in ALS patients with *C9orf72* expansions. These volcano plots illustrate the differential protein abundance in **a**, ALS patients with the pathogenic *C9orf72* repeat expansion (*n* = 29) compared to ALS patients without the expansion (*n* = 202), **b**, ALS patients with *C9orf72* (*n* = 29) compared to ALS patients without the expansion (*n* = 202) combined with control subjects (*n* = 383; comprising 169 neuro-control non-carriers, and 214 healthy control non-carriers), and **c**, asymptomatic individuals with *C9orf72* (*n* = 12) compared to symptomatic ALS patients with the expansion (*n* = 29). The dotted vertical lines indicate a ± 1.4-fold change threshold, while the dotted horizontal lines represent the 0.05 *p*-value threshold. Blue and red dots depict statistically significant down-regulated or up-regulated proteins identified through generalized linear regression. Significance was assessed using two-sided moderated t-tests with p-values adjusted by the false discovery rate (FDR) method.

We performed a comparative analysis to assess plasma protein levels in patients with ALS with *C9orf72* expansions (*n* = 29) against those lacking these expansions (*n* = 202). Our aim was to discover plasma biomarkers linked to this prevalent genetic mutation. This exploratory analysis identified eight significantly upregulated proteins in ALS *C9orf72* carriers compared with non-carriers (Extended Data Fig. 3a and Extended Data Table 3). We also compared ALS *C9orf72* carriers (*n* = 29) with non-carrier patients with ALS (*n* = 202) plus a control group (*n* = 383, which included 169 neurological control non-carriers and 214 healthy control non-carriers). The same eight proteins were elevated among ALS *C9orf72* carriers compared with controls, suggesting that these protein changes are associated with the presence of the *C9orf72* repeat expansion (Extended Data Fig. 3b and Extended Data Table 3).

We performed additional exploratory analysis to assess whether asymptomatic *C9orf72* carriers (*n* = 12) displayed a different proteomic profile compared with symptomatic *C9orf72* carriers (Extended Data Fig. 3c). Our results indicated that specific proteins (including EIF2S2, HPCAL1, JPT2, MTIF3, PDAP1 and SMAD3), which are elevated in symptomatic carriers, did not demonstrate significant changes in asymptomatic carriers and may, therefore, have potential as biomarkers for phenoconversion.

### Comparison of differentially abundant proteins in plasma to CSF

We investigated whether the proteins with differential abundance in plasma were similarly impacted in the cerebrospinal fluid (CSF). For this analysis, we extracted individual protein measurements from SomaScan data generated using CSF from 14 ALS cases and 89 healthy controls for this analysis. Variations were modeled using a generalized linear regression, adjusting for age and sex. Out of the 33 proteins, 27 were assayed on the SomaScan platform. Notably, five proteins—HSPB6, MEGF10, NEFL, RNASE3 and TPM3—demonstrated significant increases in the CSF of patients with ALS (Fig. 2d). The other proteins did not show significant differences in the CSF of patients with ALS (Table 1).

### Pathway analysis identifies skeletal muscle and neuronal processes

**Fig. 3:**
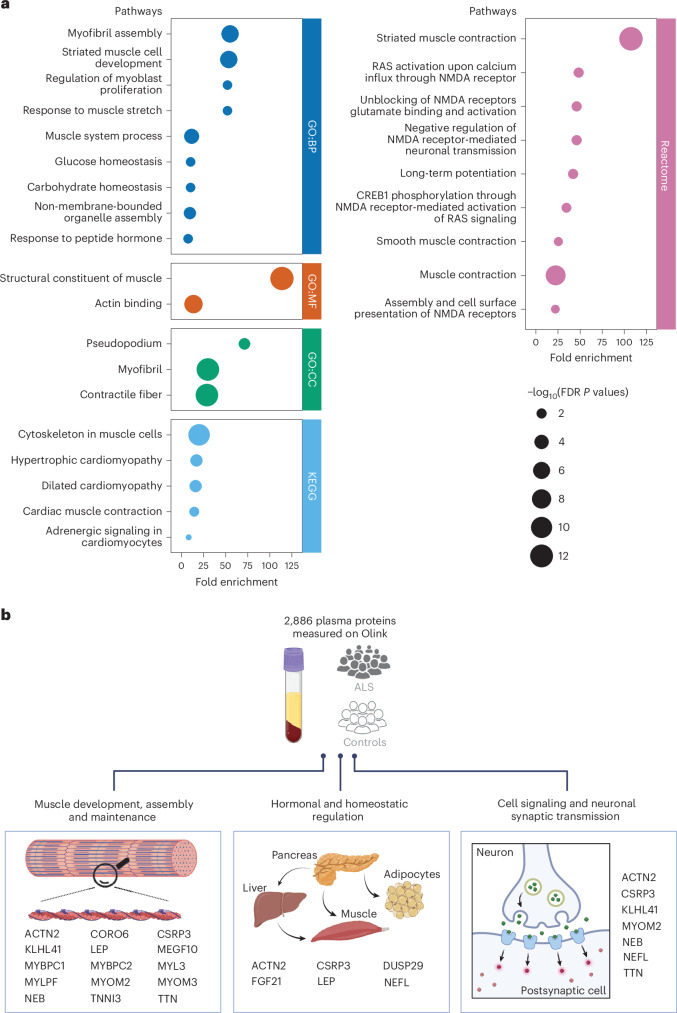
Pathway analysis of ALS based on differentially abundant plasma proteins. **a**, Functional enrichment analysis of ALS based on the differentially abundant plasma proteins (*n* = 33) performed using the ‘clusterProfiler’ software package. The *x* axis corresponds to the fold enrichment of the category in ALS cases compared with controls. A one-sided *t*-test was used to test for enrichment of genes in a particular pathway, followed by FDR correction to account for multiple testing comparisons. The size of each respective dot indicates the FDR-adjusted *P* value on a −log_10_ scale. Significant Gene Ontology (GO) enrichments for biological processes (BP, blue), molecular functions (MF, orange) and cellular functions (CC, green) as well as pathways from Reactome (magenta) and Kyoto Encyclopedia of Genes and Genomes (KEGG) (light blue) are shown. **b**, The three main BPs identified in ALS based on the enrichment analysis of the differentially abundant plasma proteins are shown. The proteins involved in each category are listed. Panels **a** and **b** created with BioRender.com.

An enrichment analysis of the 33 plasma proteins linked to ALS uncovered significant associations with multiple biological processes (Fig. 3a). Most of the identified pathways showed a strong connection with skeletal muscle and neuronal function (Fig. 3b). These results highlight the importance of cellular pathways involved in ‘skeletal muscle development and degeneration’, ‘energy metabolism’ and ‘NMDA receptor-mediated excitotoxicity’, corroborating earlier research in ALS^14–16^.

### Genetic alterations do not influence protein abundance levels

We leveraged the comprehensive multiomic data available for our cohorts to explore the relevance of the differentially abundant proteins that we observed in the ALS cases in the pathogenesis of the condition. To do this, we examined whether genetic factors affected the levels of the differentially abundant proteins. First, we searched for evidence of genetic association in the autosomal genes (*n* = 31) related to the proteins that we identified in ALS, using data from a large ALS genome-wide association study (GWAS) (*n* = 27,205 cases and *n* = 110,881 controls)^17^. None of the tested loci showed evidence of association.

Next, we implemented two-sample Mendelian randomization using merged summary-level ALS GWAS data^17^ alongside *cis*-protein quantitative trait loci (pQTL) data for the 33 plasma proteins with differential abundance in ALS^18^. This approach helps identify proteins whose levels correlate with ALS through shared causal variants. Extended Data Table 4 shows that none of the analyses reached statistical significance, reaffirming that the differential protein abundance levels seen in patients with ALS are not directly driven by inherited genetic variation encoding the levels of these proteins.

### Machine learning uncovers a distinct plasma protein signature linked to ALS

We performed supervised machine learning, hypothesizing that this method would identify a molecular signature of ALS when applied to a proteomic dataset from a well-characterized cohort. Our main goal was to create a binary classification model to distinguish patients with ALS from control individuals. The initial features used to build the machine learning model included the differentially abundant proteins (*n* = 33) and clinical parameters, including age at sample collection, sex and blood collection tube type (total number of features = 36). The system was trained on the 183 ALS cases and 309 control individuals used as the Discovery Cohort in the differential abundance analysis (now referred to as the Training Set based on standard machine learning terminology; see Fig. 1 for the analysis flowchart). We evaluated the model’s accuracy using the 48 ALS cases and 75 control individuals used as the Replication Cohort in the differential abundance analysis (now called the Testing Set).

**Fig. 4:**
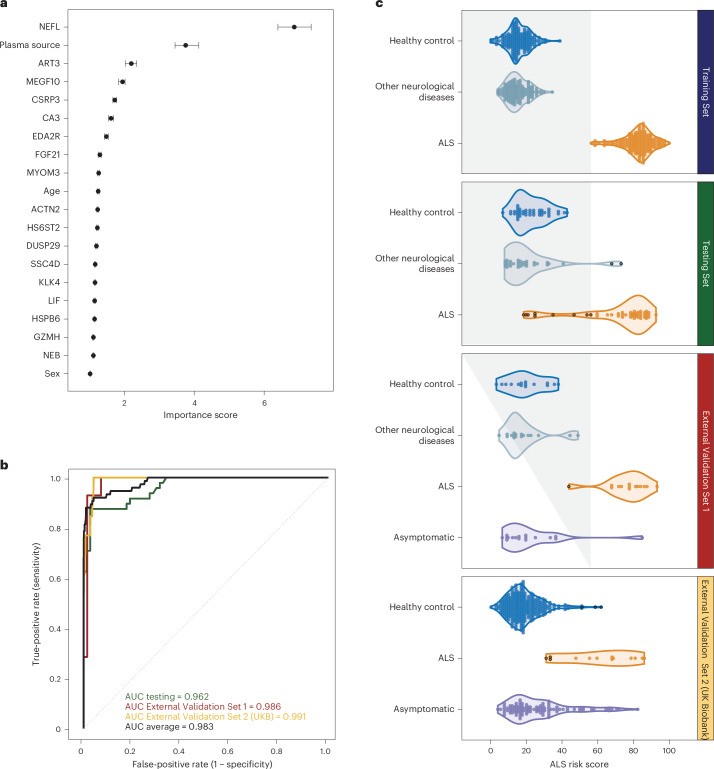
Supervised machine learning to diagnose ALS based on plasma proteins. **a**, The mean importance score of the 20 features (17 proteins plus sex, age at collection and blood collection tube type) making up the random forest model. The importance score quantifies the contribution of each feature, when permuted, to the model’s predictive performance. Features are ranked from most influential at the top (NEFL) to the least influential at the bottom (sex). Error bars represent the s.d. of the mean feature importance estimates across 100 repeated permutations. **b**, The performance of the random forest model is displayed as ROC curves. The ROC curves show the performance of the Testing Set (green, *n* = 48 ALS versus *n* = 42 healthy controls plus *n* = 32 other neurological diseases; one other neurological disease sample was excluded due to incomplete protein data), the External Validation Set 1 (red, *n* = 14 ALS versus *n* = 15 healthy controls plus *n* = 17 other neurological diseases) and the External Validation Set 2 (yellow, *n* = 13 ALS versus *n* = 23,601 healthy controls). The black curve denotes the average AUC across these three cohorts. **c**, Classification of individual samples using the ALS risk scores generated by the random forest model using the 20 features. The white area on the right denotes ALS risk scores consistent with a diagnosis of ALS. In contrast, the gray area on the left, which was manually added to the plot, delineates scores consistent with healthy control status or other neurological diseases. A black circle around a dot indicates a sample misclassified by the model.

Ten supervised machine learning algorithms were evaluated (listed in Extended Data Table 5). Of these, the random forest algorithm generated the best-performing model (Extended Data Table 5). After secondary feature selection, 20 features (17 proteins, sex, age at sample collection and plasma collection tube) were identified as the most predictive for discriminating ALS cases from healthy individuals and patients with other neurological diseases (Fig. 4a); the area under the curve (AUC) and balanced accuracy of this model were 96.2% and 89.3%, respectively, when evaluated in the Testing Set (*n* = 48 ALS cases versus *n* = 42 healthy controls plus *n* = 33 other neurological diseases) (Fig. 4b).

### The machine learning model is not powered by NEFL alone

We also examined how the exclusion of NEFL affected the predictive accuracy of the random forest model. When NEFL was removed as a feature, the area under the receiver operating characteristic curve (AUC-ROC) declined by 2.4% to 13.2% across the Testing (89.8%), External Validation Set 1 (96.2%) and External Validation Set 2 (85.9%) datasets, indicating that the model’s performance is not solely dependent on NEFL. Instead, it relies on the contributions of the other 19 features, which include 16 proteins as well as factors such as sex, age at sample collection and the type of plasma collection tube, to accurately classify samples as either ALS or control.

### Validation of the machine learning model in independent cohorts

We validated our model using two external sample sets that were not part of the training or testing process (Fig. 1). External Validation Set 1 consisted of 46 samples assayed at the National Institutes of Health (NIH), which were withheld from the primary analysis due to inadequate genetic data. External Validation Set 2 comprised Olink Explore 3072 data from the UK Biobank, including 13 ALS cases and 23,601 controls. Notably, the proteomic data for External Validation Set 1 were normalized with the same bridging samples used in the Testing Set and Training Set. In contrast, the data from External Validation Set 2 (UK Biobank) did not undergo this normalization process.

**Extended Data Fig. 4:**
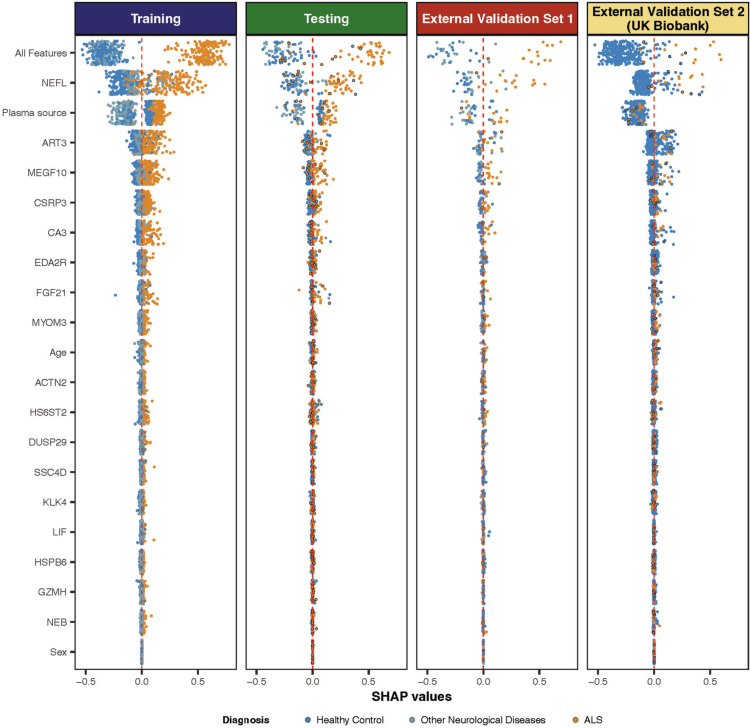
SHAP values for the features used in the supervised machine learning model to predict ALS diagnosis. Distribution of the SHAP values calculated using the machine-learning model for the 20 features with the most substantial effect on the ALS diagnosis classification model. The *Training Set* (*n* = 183 ALS cases, *n* = 172 healthy controls, and *n* = 137 patients with other neurological diseases), the *Testing Set* (*n* = 48 ALS cases, *n* = 42 healthy controls, and *n* = 33 patients with other neurological diseases; one sample from the other neurological diseases group was dropped due to incomplete protein data), the *External Validation Set 1* (*n* = 14 ALS cases, *n* = 15 healthy controls, and *n* = 17 patients with other neurological diseases), and the *External Validation Set 2* (i.e., UK Biobank, *n* = 13 ALS cases, *n* = 23,601 healthy controls) are shown. Each point represents a sample; orange dots represent ALS cases, dark blue dots represent healthy individuals, and light blue dots represent patients with other neurological diseases. A black circle around a dot indicates a sample misclassified by the model.

The AUC and balanced accuracy for the 46 samples tested at the NIH as part of the External Validation Set 1 were 99.8% and 96.4%, respectively (Fig. 4b). Despite the absence of normalization or bridging samples, External Validation Set 2 (UK Biobank) still achieved high AUC and balanced accuracy values of 99.1% and 88.1%, respectively (Fig. 4b). Overall, the supervised machine learning algorithm diagnosed 62 patients (82.7%) out of 75 ALS and correctly identified 23,548 healthy individuals (99.3%) of the 23,707 in the Testing Set, External Validation Set 1 and External Validation Set 2 (Fig. 4c and Extended Data Table 5). We then used the Shapley additive explanations (SHAP) approach to illustrate each feature’s contribution to the predictions made for each sample (Extended Data Fig. 4)^19,20^. Finally, we calculated an ALS risk score for every sample from the aggregated SHAP values, scaled between 0 and 100 using the minimum and maximum aggregated SHAP values from the Training Set samples.

We conducted additional analyses of the model’s predictive performance for a group of individuals (*n* = 355) diagnosed with neuropathy (International Classification of Diseases, 10th revision (ICD-10) codes G60–G64) or myopathy (ICD-10 codes G70–G73) using data from the UK Biobank. The random forest model successfully identified 333 (93.8%) individuals as non-ALS cases, demonstrating its capability to differentiate ALS from similar conditions.

### Estimating symptom onset using plasma proteomics

**Fig. 5:**
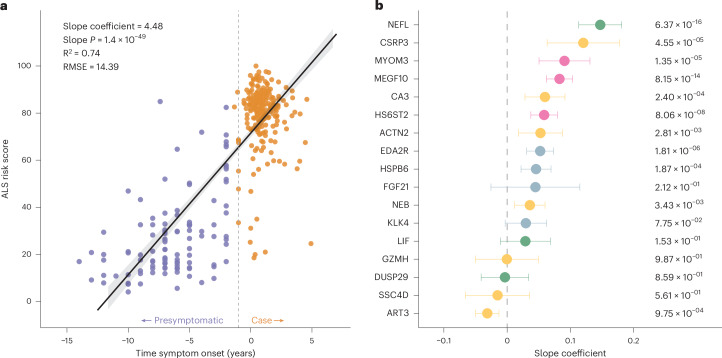
Regression analysis of ALS risk score derived from supervised machine learning predicts the age of ALS onset in asymptomatic patients. **a**, Scatter plot comparing the ALS risk score based on the random forest model and the years before symptom onset in patients with ALS. The purple dots represent plasma samples taken from presymptomatic individuals (*n* = 109 from the UK Biobank and *n* = 1 from this study) who subsequently developed ALS. The orange dots denote plasma samples taken from individuals with ALS cases in the Training Set, the Testing Set and External Validation Sets 1 and 2 (*n* = 251 ALS cases whose samples were collected within 5 years of symptom onset). The regression line with the 95% confidence interval for the mean prediction is presented as a black line with a gray band. The slope, associated *P* value, R^2^ and RMSE of the regression model are displayed. The statistical significance of each coefficient was assessed using a two-sided hypothesis test with a null hypothesis that the coefficient equals 0. The vertical dashed line indicates the rough boundary between presymptomatic individuals and those diagnosed with ALS. The data sourced from the UK Biobank are represented in whole years, explaining why some cases seem to exhibit symptoms prior to 0 years. **b**, The slope coefficients of the 17 plasma protein levels included in the random forest model when regressed individually against the time to symptom onset. Data are presented as estimated coefficient values from linear regression ± s.e., and the *P* values derived from two-sided hypothesis testing are displayed. The color of the bars denotes the Olink panel category of the protein: yellow, cardiometabolic panel; red, inflammation; green, neurology; blue, oncology.

**Extended Data Fig. 5:**
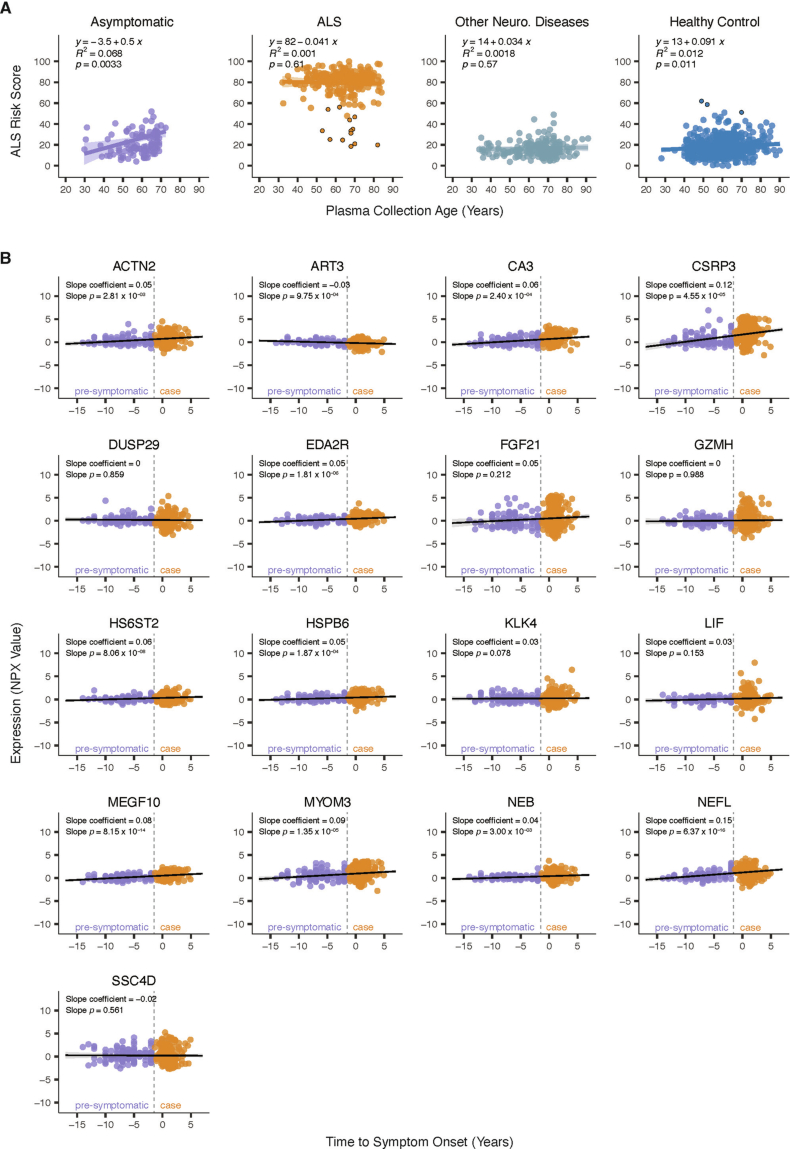
Relationship of the proteomic data to age at plasma sample collection and time to symptom onset. **a**, The distributions of the ALS Risk Score according to the age at the time of plasma collection are shown for asymptomatic individuals (purple, *n* = 126), ALS cases (orange, *n* = 259), patients with other neurological diseases (light blue, *n* = 186), and a representative sample of the healthy controls (blue, *n* = 533). Asymptomatic individuals were the only ones showing a slight correlation with time with a 5 to 14-fold steeper slope coefficient compared to healthy individuals and those with ALS or other neurological diseases. This indicates that the increase in ALS Risk Score seen in preclinical ALS is not influenced solely by normal aging or pathological aging associated with neurodegenerative diseases. The bands represent the 95% confidence intervals of the linear regression lines at each x-value. The statistical significance of each coefficient was assessed using a two-sided hypothesis test with a null hypothesis that the coefficient equals zero. The slopes, the R^2^ values, and the associated *p*-values are displayed. A black circle around a dot indicates a sample misclassified by the Random Forest model. **b**, Scatterplots comparing time to symptom onset in patients with ALS with expression levels for the 17 individual proteins included in the Random Forest model. The purple dots represent plasma samples taken from pre-symptomatic individuals (*n* = 109 from the UK Biobank, *n* = 1 from this study) who subsequently developed ALS. The orange dots denote plasma samples taken from individuals with ALS cases (*n* = 12 from the UK Biobank, *n* = 239 from this study). The gray bands represent the 95% confidence intervals of the linear regression line at each x-value. The slopes and the associated *p*-values derived from two-sided hypothesis testing are displayed.

We identified individuals (*n* = 110) who had plasma samples taken before the onset of their ALS symptoms (*n* = 109 samples from the UK Biobank and *n* = 1 sample from our cohort; mean duration from plasma sampling to symptom onset = 6.4 years, range: 2.0–14.0 years). For those individuals whose genetic data were available, 12 were confirmed to carry the *C9orf72* repeat expansion. We used these data points to examine how our machine learning model behaved prior to symptom onset. A significant association was found between the ALS risk score and the time to symptom onset, with the ALS risk score increasing progressively as patients approached symptom onset (based on *n* = 110 asymptomatic samples and *n* = 251 ALS cases that were within 5 years of symptom onset; slope coefficient = 4.48, slope *P* = 1.40 × 10^−49^, R^2^ of the regression model = 0.74 and root mean square error (RMSE) = 14.39; Fig. 5a). Notably, this increase in ALS risk score was not due to aging, as individuals with other neurological diseases, healthy individuals or even patients with ALS after they had received their diagnosis did not display the same trend (Extended Data Fig. 5a).

We investigated which proteins may be altered during the prodromal period. After adjusting for collection age, sex and plasma source, we found that 10 proteins making up the model were individually linked to the time until symptom onset when analyzing asymptomatic individuals and ALS cases together (*n* = 361) (Fig. 5b). The protein with the most significant positive coefficient was NEFL (slope coefficient = 0.147, slope *P* = 6.37 × 10^−16^), followed by CSRP3 (slope coefficient = 0.121, slope *P* = 4.55 × 10^−5^). In the analysis concentrating exclusively on patients with ALS (*n* = 251) or asymptomatic individuals (*n* = 110), only NEFL demonstrated a meaningful correlation with the time to symptom onset (Extended Data Fig. 5b). Interestingly, the slope coefficients revealed opposing trends: NEFL levels sharply increased in asymptomatic individuals as the time to onset approached zero, whereas, in patients with ALS, NEFL levels significantly decreased as the time since onset lengthened.

## Discussion

Clinicians have long sought a reliable method to diagnose ALS. Such knowledge could substantially improve patient care, assist in clinical trials and offer insights into the underlying pathogenic mechanisms^21,22^. In this study, we analyzed proteomic data from a well-characterized cohort to identify a unique molecular signature in the plasma of patients with ALS that could serve as a biomarker. We identified a panel of 33 differentially abundant proteins in ALS and validated our findings across multiple independent datasets, showing that the same protein profile can differentiate ALS from other neurological diseases, including clinically relevant differential diagnoses such as myopathies and neuropathies. We used multiomic data (that is, genetic data) from this well-characterized cohort to determine that these changes are likely secondary to the disease process. Additionally, we demonstrated that the ALS risk score computed from our machine learning model could be used as a proxy for forecasting the age at which symptoms may appear in individuals. Our data also suggest that the underlying disease process happens considerably earlier than was previously recognized. Pathway analysis showed that the relevant proteins are linked to cellular processes known to be involved in ALS^23^, confirming the effectiveness of our data-driven approach and supporting the reliability of our findings.

The key challenges in developing proteomic biomarkers for ALS have been the need for a sufficiently large dataset and the difficulty in analyzing multidimensional relationships. To address these issues, we used plasma samples from a population-based ALS registry that had enrolled patients over an extended period within a defined geographical area^9,24^. This registry collected data throughout the patientsʼ illness, providing detailed clinical information that informed our categorization efforts^19^. Our results underscore the value of disease registries in capturing the clinical heterogeneity of the ALS population^19^. The availability of multiomic data, particularly genetic data that allow for a more careful delineation of the protein signals, adds to the strength of these cohorts for research.

To date, the development of biomarkers for ALS has primarily relied on the study of single analytes, such as neurofilaments^25^. Although univariate biomarker systems hold clinical value, it is increasingly evident that they fail to capture the complexity of neurodegenerative diseases, raising concerns about their accuracy and sensitivity^25,26^. In contrast, the machine learning algorithms that we applied were adept at deciphering multifaceted relationships within data^19,27^. We generated data for almost 3,000 proteins across multiple samples to facilitate these analyses^28^. The accuracy of Olink has been validated through comparisons with proteomic measurements based on ELISA and mass spectrometry^29–34^, establishing it as an emerging standard in the field^35^. Our finding of a subset of 17 proteins (listed in Fig. 4)—attributable to multiple pathways—confirms the multiplex nature of ALS and the appropriateness of our machine learning approach for multimodal biomarker development.

We opted for plasma in our proteomic analysis due to its greater accessibility compared with CSF. Crucially, a biomarker derived from blood can facilitate early ALS evaluations, even by non-specialists^36^. Such testing may streamline referral to multidisciplinary ALS clinics, leading to earlier intervention and improved patient outcomes^37^. Additionally, biomarkers obtained through venipuncture simplify the enrollment process for clinical trials, thereby reducing costs and participant burden^38^. Examining the proteomic profile of CSF collected by lumbar puncture may incrementally improve diagnostic accuracy, as these biospecimens are in direct contact with the extracellular space of the brain and spinal cord, reflecting biochemical changes in the central nervous system^39^. Nevertheless, in neuromuscular diseases, plasma-based biomarkers may be better at measuring changes in the neuromuscular compartment than CSF, which is corroborated by our pathway analysis showing changes in the peripheral tissues. The biomarker field is evolving^40^; one possible outcome of this research is that plasma-based biomarkers could act as a screening tool for ALS, and CSF assays could provide subsequent confirmation.

We identified a unique set of plasma proteins that distinguishes ALS from healthy individuals and other neurological diseases. The NEFL protein, a proven ALS marker, is part of this panel, supporting our data-driven hypothesis-free approach^25,41^. However, the real breakthrough is the discovery of 16 additional proteins through predictive modeling, which substantially enhances ALS classification accuracy. The selection of these proteins, which have not been previously linked to ALS, highlights how proteomics and machine learning can provide fresh insights into complex diseases.

For instance, our enrichment analysis identified links between ALS and various biological pathways, particularly those involving skeletal muscle, neurons and energy metabolism. Notably, these changes may occur as many as 10 years prior to symptom onset. Several conclusions can be drawn from these findings. First, our research demonstrates that it is possible to detect the earliest stages of the disease at a molecular level. Our protein panel could act as a biomarker for this prodromal phase of ALS, similar to the biomarkers currently used in Alzheimer’s disease^42^. This capability will greatly improve clinical trials for presymptomatic patients with ALS, which typically rely on natural history studies to determine the expected rate of symptom conversion. Although there may be some noise in these measurements, longitudinal biomarker monitoring may detect an increase in their ALS risk score, indicating a molecular disease onset and predicting phenoconversion.

Second, the processes associated with the disease happen much earlier than previously thought; NEFL has been shown to increase 12 months before symptoms appear^43^. However, we discovered that the ALS risk score, determined by plasma protein levels, begins to shift in presymptomatic individuals up to 10 years before symptoms emerge, hitting the 50th percentile around 5 years prior to onset. This change in viewpoint again underscores the power of data-driven research to reveal unexpected insights into the pathophysiology of complex diseases. Our comprehensive genetic analyses revealed no evidence that genetic factors directly influence changes in plasma proteins. This suggests that the observed alterations in proteins are likely secondary effects or may originate from indirect mechanisms such as posttranscriptional or posttranslational regulation. Indeed, many of the observed changes, such as muscle regeneration and catabolic changes in energy metabolism, suggest a compensatory phase that occurs in patients with ALS long before symptom onset. However, we cannot rule out that primary muscle pathophysiology could still play an undiscovered role in the development of ALS^15^.

Our primary analysis focused on general ALS; however, we also uncovered preliminary evidence of distinct plasma proteomic profiles among ALS subpopulations. Notably, we identified eight elevated proteins in patients with a pathogenic *C9orf72* repeat expansion (Extended Data Table 3). None of these proteins was altered in the general ALS cohort, indicating that their changes may be specific to *C9orf72* carriers. These preliminary findings need confirmation in a larger sample cohort, ideally accompanied by longitudinal data, to explore how these proteins might relate to phenoconversion in *C9orf72* carriers.

### Limitations

Our study has limitations. The proteomic platform that we used quantifies approximately 3,000 proteins, which represents a substantial number of the total number of proteins in human plasma^44,45^. The unmeasured portion likely includes tissue proteins acting as markers of leakage and immunoglobulins that mirror the host’s immune response^45^, some of which may be connected to ALS. Additionally, the Olink assay cannot detect new proteins, limiting its effectiveness in investigating the role of novel proteins generated by cryptic splicing in ALS pathogenesis^46^. Future studies will investigate these broader proteomic approaches and incorporate longitudinal data collection to further validate and enhance the performance of our model.

The CSF analysis was conducted on a limited number of samples (*n* = 14 ALS cases and *n* = 89 healthy controls), which might not entirely reflect the range of protein abundance variations. However, we showed that specific proteins identified in plasma also exhibited variations in the CSF of patients with ALS. We are in the process of increasing our collection of CSF samples to facilitate further research on these and other potential proteins.

Despite the absence of shared bridging controls between our study and the UK Biobank’s Olink data, our plasma protein biomarker panel achieved over 99% accuracy in predicting the ALS or control status of 23,614 European individuals in the UK Biobank cohort. It was also able to distinguish patients with diseases such as neuropathies and myopathies that can mimic ALS. Nonetheless, a larger case–control cohort with greater ancestral diversity is necessary for additional validation to assess the global applicability of our predictive model.

In conclusion, we discovered a unique molecular profile through proteomic analysis of plasma samples from patients with ALS. This protein signature serves as a crucial factor for diagnosing ALS and distinguishes it from other neurological disorders with high specificity and sensitivity (AUC > 98%). Furthermore, the same proteomic approach enables us to predict symptom conversion in individuals who have not yet exhibited ALS symptoms. Our data-driven strategy, powered by machine learning, shows how proteomics can enhance ALS diagnosis and reshape understanding of this complex disease, suggesting that the prodromal phase happens much earlier than previously recognized.

## Methods

### Ethics and study design

This study was a cross-sectional analysis of plasma proteomic data to identify a biomarker panel for ALS. Samples were obtained from participants from the United States and Italy. Recruitment sites were located at the University of Turin in Turin, Italy; the NIH in Bethesda and Baltimore, Maryland; and Johns Hopkins University in Baltimore, Maryland.

Written consent was obtained from all individuals enrolled in this study. The institutional review boards of the National Institute on Aging (NIA) (protocol numbers 03-AG-0325 and 03-AG-N329); the National Institute of Neurological Disorders and Stroke (01-N-0206, 13-N-0188 and 17-N-0131); the National Institute of Allergy and Infectious Diseases (NIAID) (09-I-0032); Johns Hopkins University (00173663); and the University of Turin (004462) approved the study.

### Participants

From September 2008 through February 2023, 281 patients with ALS and 258 healthy individuals were enrolled in the study at the University of Turin and the NIH. The Italian samples consisted of neurologically healthy individuals (*n* = 196) and patients diagnosed with ALS (*n* = 236) living in Northern Italy and recruited in a population-based study known as the Piedmont and Valle d’Aosta Registry for ALS (PARALS; established 1 January 1995)^9^. The registry’s near-complete case ascertainment of ALS among its catchment population of almost 4.5 million inhabitants ensures the applicability of our findings^9^. The US plasma samples comprised patients diagnosed with ALS (*n* = 45) evaluated at the NIH Clinical Center in Bethesda, Maryland, as part of a natural history study (NCT03225144)^47^ and control samples collected as part of the NIH Baltimore Longitudinal Study of Aging (BLSA) (*n* = 53, NCT00233272)^48^ and at the Johns Hopkins Hospital (*n* = 9). The control samples for the CSF analyses (*n* = 89) were collected under an NIAID natural history protocol (NCT00794352).

Figure 1 shows the study workflow. The patients with ALS were diagnosed according to the revised El Escorial criteria^10^ by a neurologist specializing in ALS. Patients with familial and sporadic ALS and self-declared diverse ancestry (*n* = 3 Hispanic samples) were included in the study, ensuring a comprehensive representation of the ALS population. The control individuals were selected based on their lack of a diagnosis of ALS, neurological disease and cognitive decline in their clinical history. The control cohorts were matched to the case cohorts for race and ethnicity but not for sex or age, although the age distribution was similar (Extended Data Table 1).

We also assembled a cohort of individuals with other neurological conditions (*n* = 194, listed in Extended Data Table 1) as an additional comparison cohort. Plasma samples were collected from these participants at the NIH Clinical Center (*n* = 172) and Johns Hopkins University (*n* = 22). The other neurological disease cohort included patients diagnosed with the following conditions: corticobasal syndrome (*n* = 8 patients) was diagnosed according to the Armstrong criteria^49^; patients with Lewy body dementia (*n* = 8) were diagnosed with clinically probable disease according to consensus criteria^50,51^; the multiple system atrophy (*n* = 5) cases were diagnosed according to the Gilman criteria^52^; and Parkinson’s disease (*n* = 153) and progressive supranuclear palsy (*n* = 19) were diagnosed based on the 2015 and 2017 Movement Disorders Society criteria, respectively^53,54^. One patient was labeled as having dementia, not otherwise specified, after clinical evaluation.

### Sample collection

The plasma samples were collected via phlebotomy of the upper limb. The patients were not fasting. The Italian samples (*n* = 427) were mainly collected using heparin tubes, and the remaining samples (*n* = 306) were collected using EDTA tubes. Blood cells were removed by centrifugation within 2 hours of collection, and the supernatant, consisting of the plasma, was carefully removed without disturbing the cell pellet. All plasma samples were aliquoted and stored at −80 °C. The number of freeze–thaw cycles between aliquoting and running the proteomic assays was minimized, typically one but no more than three.

The CSF samples (*n* = 14) were collected via lumbar puncture with the patient sitting upright. A 22-gauge Sprotte spinal needle was used and inserted at the L3/L4 level. The CSF was collected in polypropylene tubes, immediately placed on ice and sent to the laboratory for processing within 30 minutes. All samples were gathered at a single location, the NIH Clinical Center in Bethesda, Maryland (NCT03109288).

### Proteomic data generation using the Olink Explore 3072 assay

For each study participant, we performed proteomic profiling on the plasma samples using the Olink Explore 3072 assay (Thermo Fisher Scientific) at the Laboratory of Clinical Investigation at the NIA, according to the manufacturer’s protocol. The Olink Explore 3072 assay quantifies 2,926 proteins (https://olink.com/products/olink-explore-3072-384) with high accuracy and specificity^11^. In brief, the platform is based on oligonucleotide-labeled antibody pairs that bind to their target protein in solution^11^. When both antibodies are close, their oligonucleotide tails hybridize and undergo extension by a DNA polymerase. This molecular process generates a unique, double-stranded DNA barcode, which is subsequently amplified and quantified using next-generation sequencing (Illumina NovaSeq).

We implemented quality control, normalization and data calibration at the probe, sample and plate levels^11^. Inter-plate sample controls (*n* = 2 per 96-well plate) were used to normalize for inter-plate variation. Proteins that did not meet the default standard Olink quality control criteria were excluded from the analysis. These steps were conducted using Olink software (version 1.0). Normalized Protein eXpression (NPX) values, representing the log_2_-transformed ratio of sample assay counts to extension control counts, were used as the final assay readout; a higher value corresponded to a higher protein expression or abundance levels^11^. Replicate samples (*n* = 3 per plate), referred to as bridging samples, were used to calculate both intra-plate and inter-plate coefficients of variation. These bridging samples are independent of the inter-plate sample controls. The coefficients of variation (%) were determined using the following formula: (s.d./mean of the replicate measurements) × 100. Samples with median NPX values (calculated from all proteins in each sample) that deviated by more than 3 s.d. from the mean of the median NPX values of all samples, as well as samples exceeding 3 s.d. from the mean of the interquartile range, were flagged as outliers and excluded from the analysis.

Olink data from the UK Biobank were obtained through the UK Biobank Research Analysis Platform (https://www.dnanexus.com/partnerships/ukbiobank). ALS cases were defined as individuals diagnosed with ICD-10 code G12.2 and confirmed at death^55^ and had their blood collected up to 1 year before symptom onset. Control samples were defined as individuals without a recorded diagnosis of myopathy (ICD-10 codes G70–G73) or neuropathy (ICD-10 codes G60–G64).

### Genetic data generation

DNA obtained from the same participants also underwent whole-genome sequencing on a HiSeq X10 sequencer (Illumina; *n* = 476, 150-bp paired-end reads, 35× coverage)^56^ or genotyping on Infinium GDA-8+ NeuroBooster BeadChips (version 1.0, Illumina; *n* = 245), according to the manufacturer’s instructions. Standard sample-level and variant-level quality control procedures were applied to the genetic datasets, and variants were extracted for the principal component analysis, performed using ‘flashPCA’ (version 2.0). Principal components 1–10 were reduced to two dimensions using UMAP version 0.2.10. These two UMAP values were used to correct for population stratification in the statistical analysis of the individual protein analytes^57^.

Although methods such as ADMIXTURE can provide percentage estimates of ancestry, the UMAP approach offers a direct and efficient way to control for confounding in our association analyses. Notably, reanalysis using all ten principal components yielded nearly identical results, confirming that the two-component UMAP adjustment sufficiently controlled for stratification in our study cohorts.

### Cohort sampling for the Discovery/Training and Replication/Testing datasets

Samples that passed Olink’s quality control were divided into two cohorts at an 80:20 ratio, labeled as the Discovery/Training and Replication/Testing datasets. The terms ‘Discovery’ and ‘Replication’ refer to differential protein abundance analysis, whereas ‘Training’ and ‘Testing’ pertain to machine learning analysis. To accurately represent cohort characteristics in the datasets, samples were matched by diagnostic groups (ALS, healthy controls and neurological controls) and the type of tube used for collection, as implemented in the ‘caret’ software package (version 6.0-94). We also balanced the selection of the Italian and United States samples across the Discovery and Replication cohorts. The Training Set included 492 individuals (*n* = 183 ALS cases, *n* = 172 healthy controls and *n* = 137 individuals with other neurological conditions). The Testing Set included 123 individuals (*n* = 48 ALS cases, *n* = 42 healthy controls and *n* = 33 individuals with other neurological diseases) (see Fig. 1 for the flowchart).

### Statistical analysis of the individual protein analytes

We performed a cross-sectional association analysis between plasma protein abundances and the diagnosis of ALS. The following generalized linear model was used to evaluate the relationship between an individual protein’s abundance and ALS status: NPX_*i*_ = *β*_1_ × ALS_*i*_ + *β*_2_ × age_*i*_ + *β*_3_ × sex_*i*_ + *β*_4_ × tubetype_*i*_ + *β*_5_ × UMAP1_*i*_ + *β*_6_ × UMAP2_*i*_, where NPX_*i*_ represents the NPX value for individual *i*; ALS_*i*_ is a binary indicator for ALS diagnosis (1 for ALS, 0 for control); age_*i*_ is the age at sample collection; sex_*i*_ is the reported sex of the individual; tubetype_*i*_ indicates the plasma collection tube type (heparin or EDTA); and UMAP1_*i*_ and UMAP2_*i*_ are the first two components of the UMAP projection of genetic data. The absence of a constant term means that there is no intercept (*β*_0_) in the model. This model was implemented using the ‘limma’ package (version 3.58.1) in R. In our analysis, all covariates were included in the model simultaneously. In alignment with our primary analysis, the regression model for the patients carrying the *C9orf72* expansion was adjusted for sex, age at sample collection, the type of plasma collection tube and the first two dimensions of UMAP based on genetic data.

Multiple comparisons were controlled using the FDR procedure defined by Benjamini and Hochberg, and two-sided FDR-adjusted *P* values are reported in Table 1, Fig. 2 and Extended Data Table 2. The significance threshold was set at an FDR *P* value of 0.05. Our Discovery Cohort, comprising 183 cases and 309 controls, had 80% power to detect a differentially abundant protein with an effect size (Cohen’s *f* statistic) of 0.242, assuming a significance threshold *P* value of 1.73 × 10^−5^. All analyses were conducted using R (version 4.3.2).

We used *z*-scores to compare the effect sizes of differentially abundant proteins in the Discovery and Replication cohorts. We used *z*-scores in this comparison because they provide an accurate statistical representation of the difference in effect size between cases and controls by taking into account the s.e. of the effect size^58^. Without standardizing with *z*-scores, the slope of the relationship between protein abundance and disease status could be artificially inflated or deflated, resulting in misleading correlation statistics.

### Comparison of Olink and ELISA for differentially abundant proteins

Quantification was performed on plasma samples (*n* = 16 ALS cases and *n* = 16 healthy controls) using commercially available colorimetric ELISA or fluorescence-based ProQuantum high-sensitivity immunoassay kits according to the manufacturer’s protocol. Immunoassay kits were available for 30 of the 33 differentially abundant proteins (Extended Data Fig. 1). The optimal dilution factor for measuring each protein was determined empirically. Each plasma sample and protein standards were assayed in duplicates. A standard curve for each protein was generated by plotting the absorbance readings at 450 nm wavelength or FAM fluorescence emission at 517 nm against the concentrations of the protein standards. A four-parameter logistic curve was fitted using GraphPad Prism software (version 10.4.1). The protein concentrations for the test samples were extrapolated from the standard curve, and the final concentrations were corrected for the dilution factor.

The Bland–Altman plot was created by calculating the differences in measurements between the ELISA or ProQuantum and Olink assays (log_2_-transformed concentration from the ELISA or ProQuantum assay minus Olink NPX) and comparing this to the mean measurement from both assays for each paired sample. This standard data visualization technique is recommended for comparing methods^59^. The 95% limits of agreement were calculated using the mean difference (bias) ± 1.96 s.d. of the differences. The relationship between the protein measurements from both assays was evaluated using the Pearson correlation coefficient. For the subset of samples (*n* = 16 ALS and *n* = 16 healthy controls), differential protein abundance analysis was performed using a generalized linear regression model with the following formula: ProteinAbundance_*i*_ = *β*1 × ALS_*i*_ + *β*2 × age_*i*_ + *β*3 × sex_*i*_ + *β*4 × tubetype_*i*_. Here, ProteinAbundance_*i*_ denotes the protein NPX value measured via Olink or the log_2_-transformed concentration from the ELISA or ProQuantum assay for individual *i*; ALS_*i*_ is a binary variable for ALS diagnosis (1 for ALS, 0 for control); age_*i*_ refers to the age at which the sample was collected; sex_*i*_ indicates the self-reported sex of the individual; and tubetype_*i*_ specifies the type of plasma collection tube (heparin or EDTA). The intercept was set to 0. All analyses and visualizations were conducted using GraphPad Prism software (version 10.4.1).

### Comparison of Olink and SomaScan data for plasma proteins

Proteomic measurements were previously generated for a subset of the BLSA control samples (*n* = 9) using SomaScan 7K, a single-stranded DNA aptamer-based proteomic platform^60^. To assess the correlation, the log_2_-transformed NPX values for the Olink 3072 Explore platform and the scaled measurements for the SomaScan 7K platform were used to calculate Spearman’s rho correlation for the 2,868 unique UniProt proteins assayed on both platforms (Extended Data Fig. 2a)^11^. The data were categorized using the *k*-means clusters identified by Katz et al.^11^ (Extended Data Fig. 2b).

### Comparison of protein in CSF and plasma

CSF proteomic measurements were generated from ALS cases (*n* = 14) and healthy control individuals (*n* = 89) using the SomaScan 7K platform. Most of the ALS cases (*n* = 13) and none of the healthy control individuals used to generate the SomaScan data were not included in the Discovery Cohort or the Replication Cohort used to create the plasma proteomic data. Individual proteins were assessed using generalized linear modeling, adjusted for age at collection and sex.

### Pathway analysis

The proteins that were differentially abundant in ALS were analyzed for pathway enrichment using ‘clusterProfiler’ (version 4.10.1) using the following databases: (1) GO^61^, (2) KEGG^62^ and (3) Reactome^63^. Pathways with a single gene were discarded, and significance was defined as an FDR-adjusted *P* value of less than 0.05.

### Regional genetic association analysis and Mendelian randomization

Regional association plots of the genes encoding the differentially abundant proteins identified in ALS cases were made with ‘locusZoomR’ (version 0.3.5) using data downloaded from a large ALS GWAS^17^. Single-nucleotide polymorphisms (SNPs) showing a *P* value less than or equal to 5.0 × 10^−8^ were regarded as evidence of genetic variants in the locus that may influence the risk of developing ALS.

We performed Mendelian randomization analyses using ‘TwoSampleMR’ (version 0.6.1)^64^. Exposures were the pQTL mapping of the plasma proteins identified by the machine learning model. These plasma pQTL profiles were obtained from GWAS data based on 54,219 UK Biobank participants, available from the Synapse web portal^18^. Only genetic variants with *P* < 1.0 × 10^−5^ from the pQTL summary statistics were included in the analysis. As the outcome, we used an ALS GWAS, comprising 29,612 cases and 122,656 control individuals^17^. To assess causality, we applied the inverse‐variance weighted method and two Mendelian randomization sensitivity tests (MR Egger and weighted median). Traits were considered consistent with a causal effect when *P* values were less than or equal to 0.05.

### Supervised machine learning

We performed supervised machine learning on the proteomic data to identify a molecular signature of ALS. To do this, we randomly selected 80% of the samples as the Training Set (*n* = 183 ALS cases versus *n* = 172 healthy controls plus *n* = 137 other neurological diseases). The remaining samples were designated as the Testing Set (*n* = 48 ALS cases versus *n* = 42 healthy controls plus *n* = 33 other neurological diseases; see Fig. 1 for flowchart). The samples in the Training Set and the Testing Set were the same as those in the Discovery Cohort and the Replication Cohort used for the differential protein abundance analysis, respectively.

We used ‘caret’ (short for ‘classification and regression training’, version 6.0-94), an open-source machine learning package that streamlines predictive model creation. The 33 differentially abundant proteins, along with clinical information, such as age at sample collection, sex and the type of blood tube used to collect the sample (total number of features = 36), were entered into the model as the starting features.

The decision to limit the feature set to 33 proteins was made to enhance the model’s stability and avoid overfitting, often seen in high-dimensional data analysis^65^. Additionally, using a minimal feature set makes the model more relevant in clinical environments, where cost and assay complexity play important roles. For instance, even though UMAP components are useful for addressing population stratification in statistical analyses, they were left out of the machine learning model. Their inclusion would require all patients to undergo genotyping, substantially increasing implementation costs and computational complexity. Consequently, we concentrated on proteins that can be directly measured in plasma, ensuring that the model remains both practical and affordable.

Ten supervised machine learning algorithms were evaluated (listed in Extended Data Table 5). Fivefold cross-validation was conducted and repeated ten times, and data were preprocessed with centering and scaling to standardize features. Adaptive resampling with a minimum of ten resamples, and a significance threshold of *α* = 0.05 using generalized least squares was employed to refine the tuning parameters for each model. Models were trained on 36 features, using the sample diagnosis as the binary target variable. The best model (random forest) was selected based on overall performance (AUC and balanced accuracy) when classifying unseen samples in the Testing Set, External Validation Set 1 and External Validation Set 2.

The random forest model was further tuned on the Training Set, and key predictive features were prioritized using the genetic algorithm feature selection method; fivefold cross-validation was conducted and repeated ten times, and data were preprocessed with centering and scaling to standardize features. The parameters used for finetuning and feature selection using the genetic algorithm method were as follows: nbits = 36, popSize = 500, maxiter = 25, pmutation = 0.1, pcrossover = 0.8 and elitism = 3. The hyperparameter for the optimized random forest model, ‘mtry’ (the number of variables considered at each split), was set to 11. For model tuning in the Discovery Cohort, the standard random forest majority-vote threshold of 0.5 was used and then directly applied to the Replication Cohort. The final model was selected based on the performance in the Testing Set, External Validation Set 1 and External Validation Set 2 cohorts.

We conducted a repeated classification analysis, excluding NEFL, to assess the impact of this protein on the model’s performance. The remaining features (that is, 16 proteins, demographic factors (sex and age at sample collection) and plasma collection tube type) were incorporated into this model.

### SHAP values and ALS risk score

SHAP values were calculated using the Shapley function from the ‘iml’ package (version 0.11.3) in R. These values reflect the contribution of each feature to the final random forest model. To determine the ALS risk score, we summed the SHAP values for all features (*n* = 20, including 17 proteins, sex, age at sample collection and type of tube used for collection) for each sample. The summed SHAP values were then scaled to a range from 0 to 100 based on the minimum and maximum values from the Training Set.

Subsequently, we conducted a generalized linear regression analysis, adjusting for age at sample collection, sex and plasma tube collection type, to assess the correlation between the time to symptom onset and (1) the ALS risk score and (2) individual protein abundances.

### Inclusion and ethics statement

All collaborators of this study who have fulfilled the criteria for authorship required by Nature Portfolio journals have been included as authors, as their participation was essential for the design and implementation of the study. Roles and responsibilities were agreed among collaborators ahead of the research. This work includes locally relevant findings, which have been determined in collaboration with local partners. This research was not severely restricted or prohibited in the researchers’ setting and did not result in stigmatization, incrimination, discrimination or personal risk to participants. Local and regional research relevant to our study was considered in citations.

### Reporting summary

Further information on research design is available in the Nature Portfolio Reporting Summary linked to this article.

## Online content

Any methods, additional references, Nature Portfolio reporting summaries, source data, extended data, supplementary information, acknowledgements, peer review information; details of author contributions and competing interests; and statements of data and code availability are available at 10.1038/s41591-025-03890-6.

## Supplementary information

Supplementary Information Supplementary Methods, consortia information and additional references for Supplementary Methods.

Reporting Summary

## Figures and Tables

**Extended Data Table 1:**
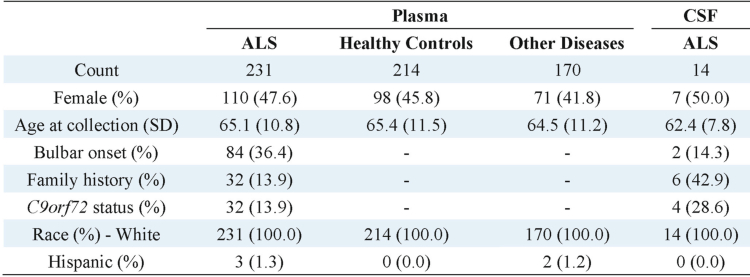
Demographic and clinical features of samples included in the proteomic analysis

The other conditions include patients with corticobasal syndrome (*n* = 6), Lewy body dementia (*n* = 7), multiple system atrophy (*n* = 5), Parkinson’s disease (*n* = 135), progressive supranuclear palsy (*n* = 16) and dementia, not otherwise specified (*n* = 1). The sex of the participants is based on self-reported sex. *C9orf72* status, carriers of the *C9orf72* pathogenic repeat expansion.

**Extended Data Table 2:**
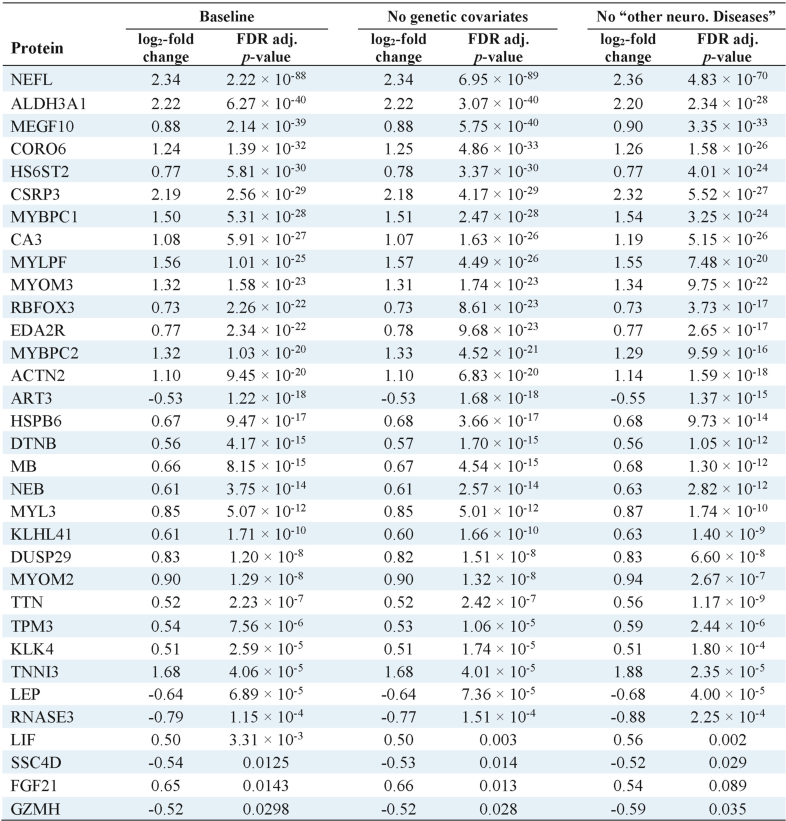
Sensitivity analyses for individual protein analytes

This table displays the linear regression analyses of the 33 proteins that are differentially expressed in ALS cases, evaluated in two scenarios: (1) the model without the genetic UMAP covariates (*n* = 183 ALS compared to *n* = 172 healthy controls and *n* = 137 patients with other neurological disorders) and (2) the model omitting samples from patients with other neurological diseases (*n* = 183 ALS compared to *n* = 172 healthy controls only). Additionally, the baseline analysis is provided for context, showing results from the linear regression analysis that incorporates genetic covariates and includes patients with other neurological diseases (*n* = 183 ALS cases compared to *n* = 172 healthy controls and *n* = 137 patients with other neurological diseases) (Table 1).

**Extended Data Table 3:**
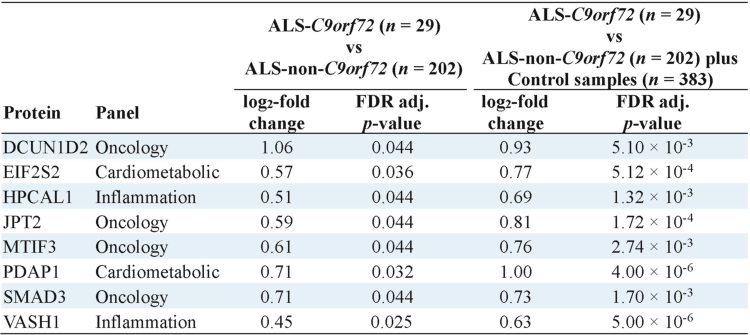
Plasma proteins associated with *C9orf72*-related ALS

This table lists the eight proteins showing differential abundance in ALS *C9orf72* carriers. Protein levels were assayed using the Olink Explore 3072 platform. The panel refers to the modular Olink panel (cardiometabolic, inflammatory and oncology) of the listed protein. Significance was assessed using two-sided moderated *t*-tests with *P* values adjusted by the FDR method.

**Extended Data Table 4:**
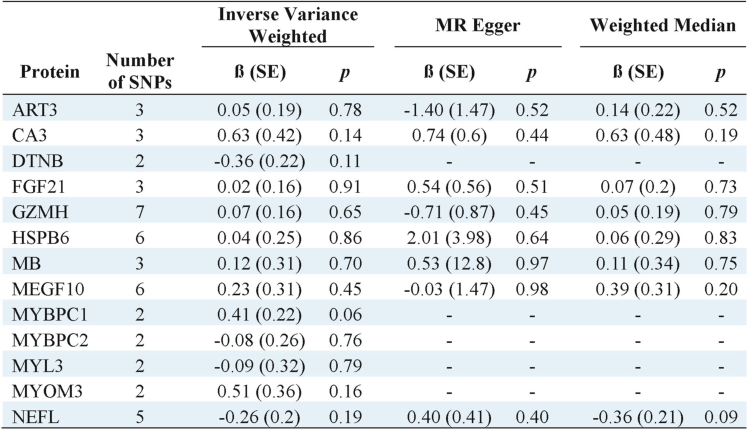
Mendelian randomization analyses performed on protein expression levels and ALS

Mendelian randomization analyses were performed using GWASs of pQTL (*n* = 54,219 UK Biobank participants) and a large ALS GWAS (*n* = 29,612 cases and *n* = 122,656 controls). This comprehensive analysis included inverse-variance weighted Mendelian randomization, MR Egger and weighted median. The proteins (*n* = 13) were analyzed when two or more SNPs were shared between the exposure and instrument GWASs. The remaining differentially expressed proteins (*n* = 20) were not evaluated due to either missing data or too few shared SNPs. Traits were considered consistent with a causal effect when *P* ≤ 0.05. β, beta; MR, Mendelian randomization. Beta estimates were derived using the Wald ratio or the inverse-variance weighted method. s.e. values were calculated using the delta method or regression residuals. Two-sided *P* values tested the null hypothesis of no causal effect.

**Extended Data Table 5:**
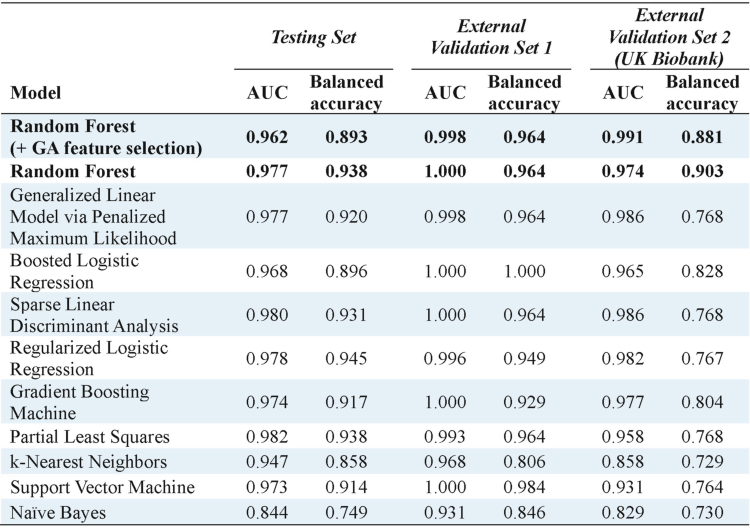
Performance of the machine learning models used to predict ALS diagnosis

This table shows the metrics used to evaluate the performance of the models based on the 36 initial features (33 proteins, sex, age at plasma collection and plasma collection tube type) across three cohorts: (1) the Testing Set (*n* = 48 ALS cases versus *n* = 42 healthy controls plus *n* = 33 patients with other neurological diseases); (2) the External Validation Set 1 (*n* = 14 ALS cases versus *n* = 15 healthy controls plus *n* = 17 patients with other neurological diseases); and (3) the External Validation Set 2/UK Biobank (*n* = 13 ALS cases versus *n* = 23,601 control samples). Additional finetuning and feature selection using the genetic algorithm method was performed for the best-performing model (random forest). The best-performing model is in bold.

## Data Availability

We made our summary statistics and individual-level proteomic data publicly available (https://zenodo.org/records/14213102)^66^. Furthermore, we developed an online tool that enables researchers to explore the machine learning model and use it with their own Olink data (https://ndru-ndrs-lng-nih.shinyapps.io/web_server_shiny/). The pQTL data from the UK Biobank used for Mendelian randomization are available at https://www.synapse.org/Synapse:syn51365301, and the ALS GWAS data are available at https://gwas.mrcieu.ac.uk/datasets/ebi-a-GCST90027163/. The Olink data from the UK Biobank can be obtained via controlled access on their web portal (https://www.ukbiobank.ac.uk/).

## Code availability

The interactive web portal was developed using ‘shiny’ (version 1.9.1). We additionally made our programming code and data publicly available to facilitate replication and enable other researchers to use our analytical pipeline in their work (https://zenodo.org/records/14213102)^66^.
