# Supplementary material for: A plasma proteomics-based candidate biomarker panel predictive of amyotrophic lateral sclerosis
Source: Nat Med. 2025 Aug 19;31(10):3440–50. doi: 10.1038/s41591-025-03890-6 (PMC12532604; doi:10.1038/s41591-025-03890-6)
Supplement: Supplementary file 1 — Supplementary Methods, consortia information and additional references for Supplementary Methods. [file 41591_2025_3890_MOESM1_ESM.pdf]

nature medicine

Supplementary information https://doi.org/10.1038/s41591-025-03890-6

A plasma proteomics-based candidate biomarker panel predictive of amyotrophic lateral sclerosis

In the format provided by the authors and unedited

SUPPLEMENTARY INFORMATION

Table of Content

Supplementary Items **Page**

Supplementary Methods
Robustness of the differentially abundant protein signals ……………………....... 2
Comparison of Olink and ELISA plasma protein measurements ……………...… 2
Comparison of Olink and Somascan plasma proteomic data …………………...... 3

Consortia Information …………………..…….…………………………………… 4

References for Supplementary Methods …………………...…….……………….... 5

SUPPLEMENTARY METHODS

Robustness of the differentially abundant protein signals

We evaluated the stability of the associations using sensitivity analyses, which confirmed that the relationships for the differentially abundant proteins ($n = 33$) remained consistent (i.e., p-value < 0.05 and same direction of effect) even after removing genetic covariates from the model. This consistency underscores the strength of these association signals (Extended Data Table 2). We conducted further analysis of differential protein abundance by excluding samples from individuals diagnosed with other neurological conditions ($n = 183$ ALS cases compared to $n = 172$ healthy controls only). All thirty-three (100.0%) of the proteins that were differentially abundant in the Discovery Cohort from the baseline analysis remained significant (i.e., p-value < 0.05) and exhibited the same direction of effect, highlighting the overall robustness of our findings (Extended Data Table 2).

Comparison of Olink and ELISA plasma protein measurements

Quantitative enzyme-linked immunosorbent assays (ELISA) were performed on 30 of the 33 differentially abundant proteins, using a subset of plasma samples ($n = 16$ ALS and $n = 16$ healthy controls) previously evaluated by Olink. Of the 30 proteins, only 16 ELISA assays had detectable levels and passed quality control. The results from these assays, along with comparisons to the corresponding Olink measurements, are presented as exploratory data in Extended Data Fig. 1. We used Pearson's correlation to evaluate the strength of the relationship between the assays and detect any linear trends in the ELISA and Olink measurements. Additionally, we employed the Bland-Altman analysis as a supplementary method to examine the alignment of the assays and detect biases, moving beyond simple correlation[1]. This statistical technique is commonly used for comparing two assays because it does not assume one to be the gold standard; rather, it assesses the differences between assays by plotting them against their averages[2].

Our Bland-Altman analysis showed that most protein pairs fell within the limits of agreement, indicating that the differences between the assays remained within expected ranges. However, consistent with previous studies[3,4], the overall Pearson's correlation was low, except for NEFL and LEP, suggesting that variations in one assay are not consistently reflected in the other. Additionally, significant differences in ELISA measurements were found for only three proteins (GZMH, beta = -2.33, p-value = 0.01; MYOM3, beta = 0.86, p-value = 6.89×10^{-3}; and NEFL, beta = 3.88, p-value = 1.20×10^{-7}). These discrepancies between most of our tested assays may arise from the different efficiencies and proteoforms recognized by antibodies specific to each assay, making direct substitution or comparison challenging. The weak correlations observed in our data indicate that further research is necessary to develop clinically viable ELISA assays for the complete protein panel.

Comparison of Olink and Somascan plasma proteomic data

A comparison of the Olink and SomaScan7K proteomic data for a subset of the same plasma samples (*n* = 9 samples, *n* = 2,868 proteins common across platforms) showed only a modest correlation (median Spearman's rho = 0.3, Extended Data Fig. 2a,b), consistent with previous reports[5,6]. Nevertheless, this comparison emphasizes that our experimental results align with those from other research laboratories[5,6]. A comparative analysis of the proteomic data from Olink and SomaScan also revealed a correlation among the differentially abundant proteins (*n* = 27 proteins shared between platforms), with most proteins (*n* = 17) showing a Spearman's rho > 0.2 (Extended Data Fig. 2c).

Other information

Consortia

The members of the American Genome Center are:

Camille Alba[11,12,13], Clifton L. Dalgard[11,12], Daniel N. Hupalo[11,13], Liqun Jiang[11,13], Elizabeth Rice[11,13], Gauthaman Sukumar[11,13]

11. Department of Anatomy, Physiology & Genetics, Uniformed Services University of the Health Sciences (USUHS), Bethesda, MD 20814, USA.
12. The American Genome Center, Collaborative Health Initiative Research Program, Uniformed Services University of the Health Sciences, Bethesda, MD 20814, USA.
13. Henry M. Jackson Foundation for the Advancement of Military Medicine, Inc., Bethesda, MD 20817, USA

REFERENCES

1. Struglics, A., Larsson, S., Lohmander, L. S. & Sward, P. Technical performance of a proximity extension assay inflammation biomarker panel with synovial fluid. *Osteoarthr Cartil Open* **4**, 100293 (2022).
2. Giavarina, D. Understanding Bland Altman analysis. *Biochem Med (Zagreb)* **25**, 141-151 (2015).
3. Vasbinder, A. et al. Assay-related differences in SuPAR levels: implications for measurement and data interpretation. *J Nephrol* **36**, 157-159 (2023).
4. Rooney, M. R. et al. Proteomic Predictors of Incident Diabetes: Results From the Atherosclerosis Risk in Communities (ARIC) Study. *Diabetes Care* **46**, 733-741 (2023).
5. Katz, D. H. et al. Proteomic profiling platforms head to head: Leveraging genetics and clinical traits to compare aptamer- and antibody-based methods. *Sci. Adv.* **8**, eabm5164 (2022).
6. Pietzner, M. et al. Synergistic insights into human health from aptamer- and antibody-based proteomic profiling. *Nat. Commun.* **12**, 6822 (2021).
