# Supplementary material for: A plasma proteomics-based candidate biomarker panel predictive of amyotrophic lateral sclerosis
Source: Nat Med. 2025 Aug 19;31(10):3440–50. doi: 10.1038/s41591-025-03890-6 (PMC12532604; doi:10.1038/s41591-025-03890-6)
Supplement: Supplementary file 2 — Reporting Summary [file 41591_2025_3890_MOESM2_ESM.pdf]

nature portfolio

Corresponding author(s): Bryan J. Traynor

Last updated by author(s): 20th June 2025

Reporting Summary

Nature Portfolio wishes to improve the reproducibility of the work that we publish. This form provides structure for consistency and transparency in reporting. For further information on Nature Portfolio policies, see our Editorial Policies and the Editorial Policy Checklist.

Statistics

For all statistical analyses, confirm that the following items are present in the figure legend, table legend, main text, or Methods section.

n/a	Confirmed	
☐	☒	The exact sample size (*n*) for each experimental group/condition, given as a discrete number and unit of measurement
☐	☒	A statement on whether measurements were taken from distinct samples or whether the same sample was measured repeatedly
☐	☒	The statistical test(s) used AND whether they are one- or two-sided<br>*Only common tests should be described solely by name; describe more complex techniques in the Methods section.*
☐	☒	A description of all covariates tested
☐	☒	A description of any assumptions or corrections, such as tests of normality and adjustment for multiple comparisons
☐	☒	A full description of the statistical parameters including central tendency (e.g. means) or other basic estimates (e.g. regression coefficient) AND variation (e.g. standard deviation) or associated estimates of uncertainty (e.g. confidence intervals)
☐	☒	For null hypothesis testing, the test statistic (e.g. *F*, *t*, *r*) with confidence intervals, effect sizes, degrees of freedom and *P* value noted<br>*Give P values as exact values whenever suitable.*
☒	☐	For Bayesian analysis, information on the choice of priors and Markov chain Monte Carlo settings
☐	☒	For hierarchical and complex designs, identification of the appropriate level for tests and full reporting of outcomes
☐	☒	Estimates of effect sizes (e.g. Cohen's *d*, Pearson's *r*), indicating how they were calculated

Our web collection on statistics for biologists contains articles on many of the points above.

Software and code

Policy information about availability of computer code

Data collection	No custom code was used to collect data for this study. Data was generated in-house using Olink assay and accession numbers forpublicly available datasets ar given the the Data section below.
Data analysis	Computational tools and code availability<br>The interactive web portal was developed using shiny (version 1.9.1). We have additionally made our programming code, summary statistics, and individual-level proteomic data publicly available to facilitate replication and enable other researchers to use our analytical pipeline in their work (https://zenodo.org/uploads/14213102).<br><br>The main software used for analysis is R (version 4.3.2). The R packages used are listed below along with the corresponding versions/release number:<br>Olink software (version 1.0), limma (version 3.58.1), umap (version 0.2.10), clusterProfiler (version 4.10.1), locusZoomR (version 0.3.5), TwoSampleMR (version 0.6.1), caret (version 6.0-94), iml (version 0.11.3), shiny (version 1.9.1)<br><br>Additional softwares used that are not part of R: GraphPad Prism software (version 10.4.1), flashPCA (version 2.0)

For manuscripts utilizing custom algorithms or software that are central to the research but not yet described in published literature, software must be made available to editors and reviewers. We strongly encourage code deposition in a community repository (e.g. GitHub). See the Nature Portfolio guidelines for submitting code & software for further information.

Data

Policy information about availability of data

All manuscripts must include a data availability statement. This statement should provide the following information, where applicable:

- Accession codes, unique identifiers, or web links for publicly available datasets
- A description of any restrictions on data availability
- For clinical datasets or third party data, please ensure that the statement adheres to our policy

Data availability
We have made our summary statistics and individual-level proteomic data publicly available (see https://zenodo.org/uploads/14213102). Furthermore, we have developed an online tool that enables researchers to explore the machine-learning model and utilize it with their own Olink data (https://ndru-ndrs-lng-nih.shinyapps.io/web_server_shiny/). The pQTL data from the UK Biobank used for Mendelian randomization are available at www.synapse.org/ Synapse:syn51365301, and the ALS GWAS data are available at https://gwas.mrcieu.ac.uk/datasets/ebi-a-GCST90027163/. The Olink data from the UK Biobank can be obtained via controlled access on their web portal (www.ukbiobank.ac.uk).

Proteomic data generation
For each study participant, we performed proteomic profiling on the plasma samples using the Olink Explore 3072 Assay. We implemented quality control, normalization, and data calibration at the probe, sample, and plate levels. These steps were conducted using the Olink software (version 1.0).

Genetic data generation
DNA obtained from the same participants underwent whole-genome sequencing on a HiSeq X10 sequencer (Illumina, San Diego, n = 476, 150-base paired-end reads, 35x coverage) or genotyping on Infinium GDA-8+ NeuroBooster BeadChips (version 1.0, Illumina, n = 245). Standard sample-level and variant-level quality-control procedures were applied to the genetic datasets, and variants were extracted for the principal component analysis, performed using flashPCA (version 2.0).

Research involving human participants, their data, or biological material

Policy information about studies with human participants or human data. See also policy information about sex, gender (identity/presentation), and sexual orientation and race, ethnicity and racism.

Reporting on sex and gender	No sex-specific analyses were performed. Biological sex, as reported by the participant, was used as a covariate in the following analyses: (1) a generalized linear model to identify the association between plasma protein abundances and the diagnosis of ALS, (2) supervised machine learning performed on the proteomic dataset to identify a molecular signature of ALS, (3) Shapley additive explanations (SHAP) analysis to quantify the contribution of each feature in the model (including sex of the participant) to the final Random Forest model.
Reporting on race, ethnicity, or other socially relevant groupings	No population-specific analyses were performed. The race and ethnicity of the participants is reported in the table detailing the demographics of the cohorts included in the study. Variants were extracted from the genetic data generated for the participants and used for the principal component analysis. This analysis was performed using flashPCA (version 2.0). Principal components 1–10 were reduced to two dimensions using UMAP (Uniformed Manifold Approximation, version 0.2.10). These two UMAP values were used to correct for population stratification in the statistical analysis of the individual protein analytes.
Population characteristics	The covariate-relevant population characteristics of the human research participants are summarized in Extended Data Table 1. Specifically, n = 110 (47.6%) of the ALS cases, n = 98 (45.8%) of the healthy control participants, and n = 71 (41.8%) of participants with other neurological diseases were female. The average age at collection was 65.1 (standard deviation = 10.8) for ALS cases, 65.4 (11.5) for healthy control participants, and 64.5 (11.2) for participants with other neurological diseases. All ALS cases, healthy control participants, and participants with other neurological diseases were White. Among them, 3 (1.3%) of the ALS cases and 2 (1.2%) of the participants with other neurological diseases were Hispanic.
Recruitment	The study was a cross-sectional analysis of plasma proteomic data to identify a biomarker panel for ALS. Samples were obtained from participants from the United States and Italy. Recruitment sites were located at the University of Turin in Turin, Italy, the U.S. National Institutes of Health in Bethesda and Baltimore, Maryland, USA, and Johns Hopkins University in Baltimore, Maryland, USA. From September 2008 through February 2023, 281 patients with ALS and 258 healthy individuals were enrolled in the study at the University of Turin, Italy, and the U.S. National Institutes of Health (NIH). The Italian samples consisted of neurologically healthy individuals (n = 196) and patients diagnosed with ALS (n = 236) living in Northern Italy and recruited in a population-based study known as the Piedmont and Valle d'Aosta Registry for ALS (PARALS; established January 1, 1995). The registry's near-complete case ascertainment of ALS among its catchment population of almost 4.5 million inhabitants ensures the applicability of our findings. The U.S. plasma samples comprised patients diagnosed with ALS (n = 45) evaluated at the NIH Clinical Center, Bethesda, Maryland, as part of a natural history study (Clinical Trials number: NCT03225144), and control samples collected as part of the NIH Baltimore Longitudinal Study of Aging (n = 53, NCT00233272) and at the Johns Hopkins Hospital (n = 9). The control samples for the CSF analyses (n = 89) were collected under an NIAID natural history protocol (Clinical Trials number: NCT00794352). The ALS patients were diagnosed according to the revised El Escorial criteria by a neurologist specializing in ALS. Patients with familial and sporadic ALS and self-declared diverse ancestry (n = 3 Hispanic samples) were included in the study. The control subjects were selected based on their lack of a diagnosis of ALS, neurological disease, and cognitive decline in their clinical history. The control cohorts were matched to the case cohorts for race and ethnicity but not for sex or age, although the age distribution was comparable (Table S1).<br>We also assembled a cohort of individuals with other neurological conditions (n = 194, listed in Extended Data Table 1) as an additional comparison cohort. Plasma samples were collected from these participants at the NIH Clinical Center (n = 172) and Johns Hopkins University (n = 22). The other neurological disease cohort included patients diagnosed with the following conditions: Corticobasal syndrome (n = 8 patients) was diagnosed according to the Armstrong criteria. The patients with Lewy body dementia (LBD, n = 8) were diagnosed with clinically probable disease according to consensus criteria. The multiple

system atrophy (MSA, n = 5) cases were diagnosed according to the Gilman criteria. Parkinson's disease (n = 153) and progressive supranuclear palsy (n = 19) were diagnosed based on the 2015 and 2017 Movement Disorders Society criteria, respectively. One patient was labeled as having dementia, not otherwise specified after clinical evaluation.

Notes on potential recruitment bias: The most significant recruitment bias is that most samples collected and included in the analysis are of European ancestry. Thus, results from this will have to be validated in other non-European centric cohorts to assess the broader applicability of our findings.

Ethics oversight

Written consent was obtained from all individuals enrolled in this study. The institutional review boards of the National Institute on Aging (protocol numbers 03-AG-0325 and 03-AG-N329), National Institute of Neurological Disorders and Stroke (01-N-0206, 13-N-0188, and 17-N-0131), National Institute of Allergy and Infectious Diseases (09-I-0032), Johns Hopkins University (00173663), and the University of Turin (004462) approved the study.

Note that full information on the approval of the study protocol must also be provided in the manuscript.

Field-specific reporting

Please select the one below that is the best fit for your research. If you are not sure, read the appropriate sections before making your selection.

☒ Life sciences ☐ Behavioural & social sciences ☐ Ecological, evolutionary & environmental sciences

For a reference copy of the document with all sections, see nature.com/documents/nr-reporting-summary-flat.pdf

Life sciences study design

All studies must disclose on these points even when the disclosure is negative.

Sample size

Our discovery cohort, comprising 183 cases and 309 controls, had 80% power to detect a differentially abundant protein with an effect size (Cohen's f statistic) of 0.242, assuming a significance threshold p-value of 1.73 × 10-5.

Data exclusions

Proteins that did not meet the default standard Olink quality control criteria were excluded from the analysis. These steps were conducted using the Olink software (version 1.0). These exclusions are standard in the field.

Replication

Overall results from our replication efforts: Results from the differential abundance was replicated in an independent cohort. The machine learning classifier model was fully tested and validated in two external datasets.

Figure 1 gives an overview of the study and analysis. For the differential abundance analysis, we randomly designated 80% of the samples as the Discovery Cohort (n = 183 ALS cases versus n = 173 healthy controls plus n = 137 other neurological diseases). The remaining samples constituted the Replication Cohort (n = 48 ALS cases versus n = 42 healthy controls plus n = 33 other neurological diseases). We balanced the selection of the Italian and United States samples across the Discovery and Replication Cohorts.

For the machine learning, the system was trained on the 183 ALS cases and 309 control subjects used as the Discovery Cohort in the differential abundance analysis (referred to as the Training Set based on standard machine learning terminology). We evaluated the models' accuracy using the 48 ALS cases and 75 control subjects used as the Replication Cohort in the differential abundance analysis (now called the Testing Set).
We validated our machine learning model using two external sample sets that were not part of the training or testing process. External Validation Set 1 consisted of 46 samples assayed at NIH, which were withheld from the primary analysis due to inadequate genetic data. External Validation Set 2 comprised Olink Explore 3072 data from the UK Biobank, including 13 ALS cases and 23,601 controls. Notably, the proteomic data for External Validation Set 1 were normalized with the same bridging samples used in the Testing Set and Training Set. In contrast, the data from External Validation Set 2 (UK Biobank) did not undergo this normalization process.

Randomization

For the differential abundance analysis, we randomly designated 80% of the samples as the Discovery Cohort (n = 183 ALS cases versus n = 173 healthy controls plus n = 137 other neurological diseases). The remaining samples constituted the Replication Cohort (n = 48 ALS cases versus n = 42 healthy controls plus n = 33 other neurological diseases). We balanced the selection of the Italian and United States samples across the Discovery and Replication Cohorts.

Blinding

The operators generating the Olink data were blinded to the case-control status. The analysts were not blinded to the case-control status. Blinding of the samples was exercised to prevent operator bias when performing the assay. The analyst was not blinded to the analysis as the supervised machine learning model requires the actual label of the case-control status for each sample be known so that the model accuracy can be assessed.

Reporting for specific materials, systems and methods

We require information from authors about some types of materials, experimental systems and methods used in many studies. Here, indicate whether each material, system or method listed is relevant to your study. If you are not sure if a list item applies to your research, read the appropriate section before selecting a response.

Materials & experimental systems

n/a	Involved in the study
☐	☒ Antibodies
☒	☐ Eukaryotic cell lines
☒	☐ Palaeontology and archaeology
☒	☐ Animals and other organisms
☐	☒ Clinical data
☒	☐ Dual use research of concern
☒	☐ Plants

Methods

n/a	Involved in the study
☒	☐ ChIP-seq
☒	☐ Flow cytometry
☒	☐ MRI-based neuroimaging

Antibodies

Antibodies used

We performed proteomic profiling on the plasma samples using the Olink Explore 3072 Assay (Thermo Fisher Scientific Inc., Waltham, MA) at the Laboratory of Clinical Investigation at the National Institute on Aging (Baltimore, MD), according to the manufacturer's protocol. The Olink Explore 3072 assay quantifies 2,926 proteins (https://olink.com/products/olink-explore-ht) with high accuracy and specificity. Briefly, the platform is based on oligonucleotide-labeled antibody pairs that bind to their target protein in solution. When both antibodies are close, their oligonucleotide tails hybridize and undergo extension by a DNA polymerase. This molecular process generates a unique, double-stranded DNA barcode, which is subsequently amplified and quantified using next-generation sequencing (Illumina NovaSeq). The antibodies used by the Olink system are proprietary.
We also measured protein abundance in plasma samples using commercially available colorimetric ELISA or fluorescence-based ProQuantum immunoassay kits according to the manufacturer's protocol. Thirty of the 33 proteins with differential abundance in our primary analysis were tested. The antibodies used in each of the kits are proprietary.

Validation

Not applicable.

Clinical data

Policy information about clinical studies
All manuscripts should comply with the ICMJE guidelines for publication of clinical research and a completed CONSORT checklist must be included with all submissions.

Clinical trial registration

The U.S. plasma samples comprised patients diagnosed with ALS (n = 45) evaluated at the NIH Clinical Center, Bethesda, Maryland, as part of a natural history study (Clinical Trials number: NCT03225144), and control samples collected as part of the NIH Baltimore Longitudinal Study of Aging (n = 53, NCT00233272) and at the Johns Hopkins Hospital (n = 9). The control samples for the CSF analyses (n = 89) were collected under an NIAID natural history protocol (Clinical Trials number: NCT00794352).

Study protocol

The study protocol details of clinical trial NCT03225144 can be found at https://clinicalstudies.info.nih.gov/ProtocolDetails.aspx?id=2017-N-0131 (accessed 26 February 2025). The study protocol details of clinical trial NCT00233272 can be found at https://clinicalstudies.info.nih.gov/protocoldetails.aspx?id=03-AG-0325&&query= (accessed 26 February 2025). The study protocol details of clinical trial NCT00794352 can be found at https://clinicalstudies.info.nih.gov/ProtocolDetails.aspx?id=09-I-0032 (accessed 26 February 2025).

Data collection

From September 2008 through February 2023, 281 patients with ALS and 258 healthy individuals were enrolled in the study at the University of Turin, Italy, and the U.S. National Institutes of Health (NIH). The Italian samples consisted of neurologically healthy individuals (n = 196) and patients diagnosed with ALS (n = 236) living in Northern Italy and recruited in a population-based study known as the Piedmont and Valle d'Aosta Registry for ALS (PARALS; established January 1, 1995). The U.S. plasma samples comprised patients diagnosed with ALS (n = 45) evaluated at the NIH Clinical Center, Bethesda, Maryland, as part of a natural history study (Clinical Trials number: NCT03225144), and control samples collected as part of the NIH Baltimore Longitudinal Study of Aging (n = 53, NCT00233272) and at the Johns Hopkins Hospital (n = 9). The control samples for the CSF analyses (n = 89) were collected at the NIH Clinical Center under an NIAID natural history protocol (Clinical Trials number: NCT00794352).

Outcomes

These were natural history studies, and therefore outcomes are not applicable in this context.

Plants

Seed stocks	Not applicable.
Novel plant genotypes	Not applicable.
Authentication	Not applicable.
